# Polar Flagella Glycosylation in *Aeromonas*: Genomic Characterization and Involvement of a Specific Glycosyltransferase (Fgi-1) in Heterogeneous Flagella Glycosylation

**DOI:** 10.3389/fmicb.2020.595697

**Published:** 2021-01-18

**Authors:** Gabriel Forn-Cuní, Kelly M. Fulton, Jeffrey C. Smith, Susan M. Twine, Elena Mendoza-Barberà, Juan M. Tomás, Susana Merino

**Affiliations:** ^1^Departamento de Genética, Microbiología y Estadística, Sección Microbiología, Virología y Biotecnología, Facultad de Biología, Universidad de Barcelona, Barcelona, Spain; ^2^National Research Council Canada, Human Health Therapeutics Research Centre, Ottawa, ON, Canada; ^3^Faculty of Science, Carleton University, Ottawa, ON, Canada

**Keywords:** *Aeromonas*, polar flagellum, motility, glycosylation island, glycosyltransferases

## Abstract

Polar flagella from mesophilic *Aeromonas* strains have previously been shown to be modified with a range of glycans. Mass spectrometry studies of purified polar flagellins suggested the glycan typically includes a putative pseudaminic acid like derivative; while some strains are modified with this single monosaccharide, others modified with a heterologous glycan. In the current study, we demonstrate that genes involved in polar flagella glycosylation are clustered in highly polymorphic genomic islands flanked by pseudaminic acid biosynthetic genes (*pse*). Bioinformatic analysis of mesophilic *Aeromonas* genomes identified three types of polar flagella glycosylation islands (FGIs), denoted Group I, II and III. FGI Groups I and III are small genomic islands present in *Aeromonas* strains with flagellins modified with a single monosaccharide pseudaminic acid derivative. Group II were large genomic islands, present in strains found to modify polar flagellins with heterogeneous glycan moieties. Group II, in addition to *pse* genes, contained numerous glycosyltransferases and other biosynthetic enzymes. All Group II strains shared a common glycosyltransferase downstream of *luxC* that we named flagella glycosylation island 1, *fgi-1*, in *A. piscicola* AH-3. We demonstrate that Fgi-1 transfers the first sugar of the heterogeneous glycan to the pseudaminic acid derivative linked to polar flagellins and could be used as marker for polysaccharidic glycosylation of *Aeromonas* polar flagella.

## Introduction

Protein glycosylation is a common post-translational modification in bacteria and has been described in both gram-negative and gram-positive bacteria (De Maayer and Cowan, 2016; Schäffer and Messner, 2017). This post-translational modification has been identified in many target proteins, including surface proteins such as pili, flagella, adhesins and surface layer proteins (Benz and Schmidt, 2002; Abu-Qarn et al., 2008). Glycoproteins are reported to play roles in adhesion, proteins stabilization, motility and evasion of immune responses, however in many cases, the precise function of the glycan modification has not been determined (Szymanski and Wren, 2005; Tan et al., 2015). Bacterial glycans show high variability in structures and composition (Benz and Schmidt, 2002), and are linked to the amide group of asparagine residues (*N*-glycosylation) or to the hydroxyl group of serine (Ser) or threonine (Thr) residues (*O*-glycosylation) (Nothaft and Szymanski, 2010).

Aeromonads are rod-shaped, Gram-negative bacteria, ubiquitous in the environment, and frequently associated with fresh or estuarine water. They are emerging as the causative agents of gastrointestinal and extraintestinal disease in a vast evolutionary range of animals (Janda and Abbott, 2010). Although the most common complications from these pathogens are easily tractable, the number of reported infections caused by these microorganisms in humans has been steadily rising in recent years (Igbinosa et al., 2012). *Aeromonas* infections present a serious threat to the increasing population of immunocompromised patients, causing severe septicaemia, and death (Parker and Shaw, 2011). The pathogenicity of Aeromonads is multifactorial, and depends on specific strain characteristics, but common pathogenic factors across *Aeromonadaceae* are toxins and secretion systems, outer-membrane proteins, capsules, polysaccharides, cell-wall proteins and flagella (Tomás, 2012).

Mesophilic *Aeromonas* have a single polar flagellum which is produced constitutively. In addition, 50–60% of clinical isolates also express a lateral inducible flagella (Gavín et al., 2002). Polar flagellins, the structural protein of the flagellar filament, of strains analyzed to date are reported to be *O-*glycosylated at 5–8 Ser or Thr residues of the central immunogenic D2/D3 domains (Tabei et al., 2009; Wilhelms et al., 2012; Fulton et al., 2015). However, the observed glycans show diversity in their carbohydrate composition and chain length between strains. In *Aeromonas caviae* Sch3N and *Aeromonas hydrophila* AH-1, the glycan modifying polar flagellin was observed to be a single monosaccharide, which was speculated to be a pseudaminic acid derivative (Tabei et al., 2009; Fulton et al., 2015); in *Aeromonas piscicola* AH-3, the glycan modifying polar flagellin was reported to be a heptasaccharide comprised of one putative pseudaminic acid derivative, three N-acetylhexosamines (HexNAc), two hexoses (Hex) and one unknown glycan of 102 Da (Wilhelms et al., 2012). In all strains, a putative pseudaminic acid derivative was found to be the linking sugar that directly modifies the Ser or Thr residue. Regardless of glycan composition, the post-translational modification has been shown to be required for the correct polar flagellum assembly in all *Aeromonadaceae* with characterized glycosylation to date (Parker et al., 2012; Merino and Tomás, 2014; Fulton et al., 2015) and is essential for adhesion, biofilm formation and colonization (Merino et al., 1999; Rabaan et al., 2001; Gavín et al., 2002).

**Figure 1:**
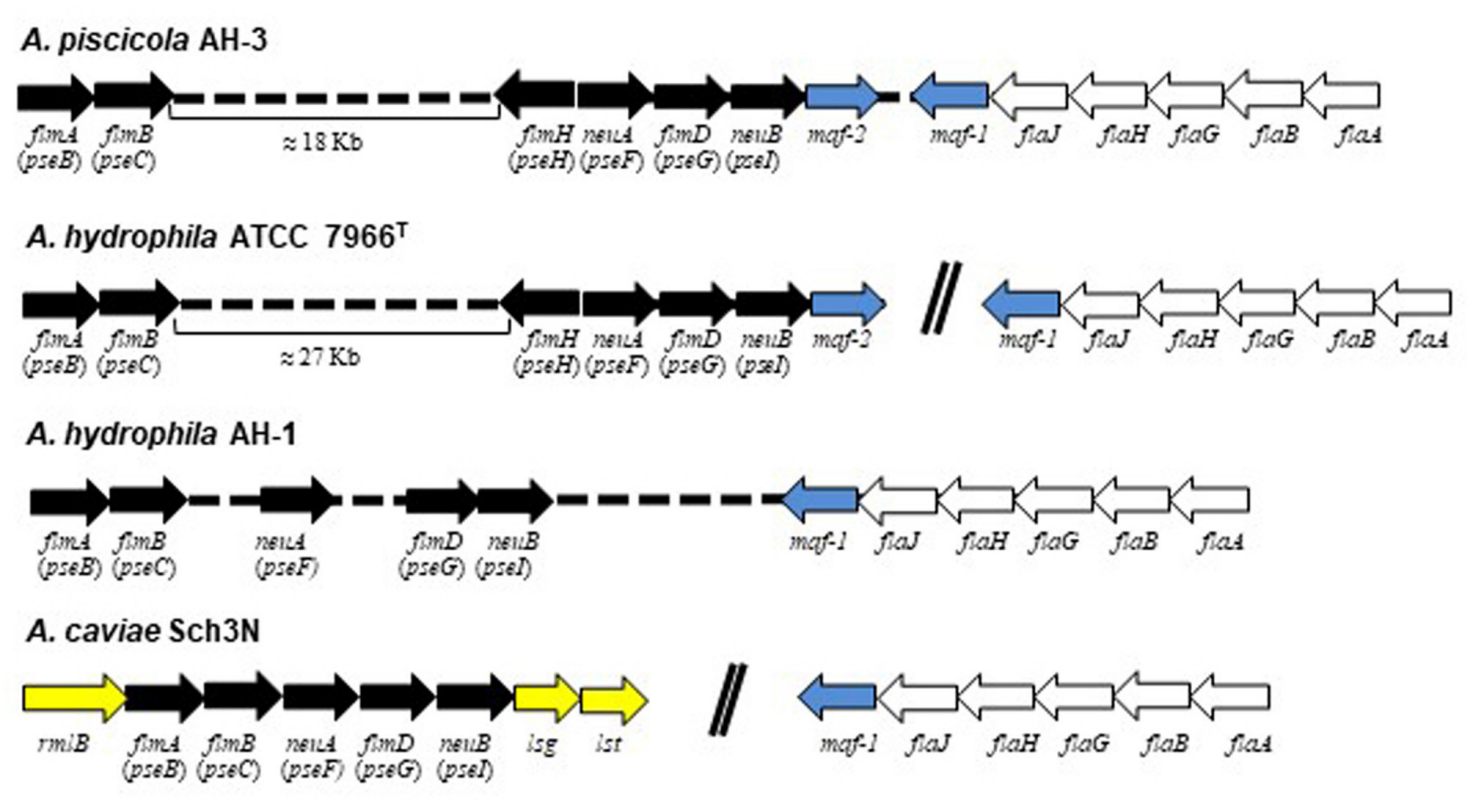
Comparative schematic representation of *A. piscicola* AH-3*, A. hydrophila* ATCC7966^TM^*, A. hydrophila* AH-1 and *A. caviae* Sch3N pseudaminic acid biosynthetic genes *flmA (pseB), flmB (pseC), flmH* (*pseH), neuA* (*pseF), flmD* (*pseG)*, and *neuB* (*pseI)*, also known as the *flm* locus, in black; *maf* glycosiltransferases in blue; polar flagella genes in white; and O-antigen LPS genes in yellow. Arrows of the same color indicates homologous genes among these bacteria.

In many bacteria, the sugar biosynthetic pathways and associated glycosyltransferases required for flagellin modification are encoded in close proximity to the genes encoding the flagellar apparatus. In *Aeromonas*, the pseudaminic acid biosynthesis locus, formed by PseB, PseC, PseF, PseG, and PseI (also known as the *flm* locus, or *flmA, flmB, neuA*-like, *flmD*, and *neuB*-like, respectively), is reported to exist in two different chromosomal locations (Canals et al., 2007; Tabei et al., 2009). In *A. caviae* Sch3N these genes are located adjacent to the O-antigen lipopolysaccharide (LPS) biosynthesis locus and its mutation abolishes both O-antigen LPS and polar flagellum formation (Tabei et al., 2009). However, in *A. hydrophila* AH-1 and *A. piscicola* AH-3 many of these genes are adjacent to the polar flagella region 2, which contains the polar flagellin genes. Mutations in this region have been reported only to affect the flagella formation (Canals et al., 2007; Fulton et al., 2015). Furthermore, in *A. caviae* Sch3N the glycosyltransferase *maf-1*, located adjacent to the polar flagella region 2, was identified as the prime candidate to transfer the pseudaminic acid derivative to the flagellin monomers prior to their deliver to the flagella export apparatus (Parker et al., 2012, 2014). Homologous and identically localized glycosyltransferases were found in *A. hydrophila* AH-1 (AO056-RS01190) and *A. piscicola* AH-3 (*maf-1*), which are also adjacent to the pseudaminic acid biosynthetic genes (Canals et al., 2006; Forn-Cuní et al., 2016a,b) (Figure 1).

In this study, we analyze and classify the *Aeromonadaceae* flagella glycosylation islands (*fgi*), across all the publicly available mesophilic *Aeromonas* genomes and characterize for the first time a common but unstudied glycosyltransferase, Fgi-1, in strains with heterogeneous glycans, as marker for polysaccharidic glycosylation of *Aeromonas* polar flagellum.

## Materials and Methods

### Identification and Characterization of Polar Flagellin Glycosylation Islands (*fgi*)

The genome sequences of 265 mesophilic *Aeromonads* strains were retrieved from the NCBI database regardless of their assembly completeness. To locate the pseudaminic acid biosynthesis cluster, we performed a local *tblastn* of the *A. piscicola* AH-3 PseI and PseC in each genome. We retained 50 complete genomes and a further 160 genomes in which both genes were present in the same contig or scaffold, independently of the space between the two genes. Genes on the selected region were predicted with Glimmer v3.0.2 (Delcher et al., 2007), and explored using Sybil (Riley et al., 2012) as implemented in the CloVR Comparative Pipeline (Angiuoli et al., 2011). GC percentage was calculated based on the sequences of the species *A. piscicola* AH-3, *A. hydrophila* ATCC7966^T^ and AH-1. All the following bioinformatic analysis with at least one of each three clusters, when not mentioned, were based on AH-3. Alignment of the 97 Fgi-1 protein sequences was performed by ClustalW and the percentage of identity between them was calculated using the Clustal Omega service (Sievers et al., 2011) of the EMBL-EBI website (Li et al., 2015). Protein domains were determined with the NCBI Conserved Domain Database (CDD) (Marchler-Bauer et al., 2014), and the SMART sequence analysis (Letunic et al., 2014). The presence of signal peptides was explored with the SignalP 4.1 Server (Nielsen, 2017) and of transmembrane helices using the TMHMM Server v2.0 (Krogh et al., 2001). The tertiary structure of Fgi-1 was modeled with the Intensive method from the Phyre2 Protein Fold Recognition Server (Kelley et al., 2015) and the I-Tasser Suite (Yang et al., 2014). Finally, the Ligand Binding Sites of the Phyre2 model were predicted with 3DLigandSite (Wass et al., 2010).

### Bacterial Strains, Plasmids and Growth Conditions

Bacterial strains and plasmids used in this study are listed in Table 1. *E. coli* strains were grown on Luria-Bertani (LB) Miller broth and LB Miller agar at 37°C. *Aeromonas* strains were grown either in tryptic soy broth (TSB) or agar (TSA) at 25°C. When required, chloramphenicol (25 μg/ml), rifampicin (100 μg/ml) and spectinomycin (50 μg/ml) were added to media. Media were supplemented with 0.2% (w/v) L-arabinose to induce recombinant protein expression under the arabinose promoter on pBAD33.

**Table 1:** Bacterial strains and plasmid used in this study.

**Strain or plasmid**	**Genotype and/or phenotype^**a**^**	**References**
**Strains**		
*A. hydrophila*		
AH-3	*A. piscicola* wild type	Forn-Cuní et al., 2016b
ATCC7966^T^	*A. hydrophila* wild type	Seshadri et al., 2006
HM21	*A. veronii* wild type	Bomar et al., 2013
AH-405	AH-3, spontaneous Rif^r^	Altarriba et al., 2003
ATCC7966-Rif	ATCC7966^T^, spontaneous Rif^r^	Merino and Tomás, 2016
AH-3ΔFgi	AH-405, Δ*fgi-1-12*	This work
AH-3ΔFgi*-*1	AH-405, Δ*fgi-1*	This work
ATCCΔAHA4171	ATCC7966-Rif; ΔAHA_4171	This work
*E. coli*		
DH5α	F^−^*endA hdsR17*(rk^−^ mk^+^) *supE44 thi-1 recA1 gyr-A96* ϕ80*lacZ*	Hanahan, 1983
MC1061λpir	*thi thr1 leu6 proA2 his4 argE2 lacY1 galK2 ara14 xyl5 supE44* λ *pir*	Rubires et al., 1997
**Plasmids**		
pRK2073	Helper plasmid, Sp^r^	Rubires et al., 1997
pDM4	Suicide plasmid, *pir* dependent with *sacAB* genes, oriR6K, Cm^r^.	Milton et al., 1996
pDM-AH3Fgi	pDM4Δ*fgi-1-12* of AH-3, Cm^r^.	This work
pDM-Fgi-1	pDM4Δ*fgi-1* of AH-3, Cm^r^.	This work
pDM-AHA4171	pDM4ΔAHA_4171 of ATCC7966^T^, Cm^r^.	This work
pBAD33	pBAD33 arabinose-induced expression vector with Cm^r^	Guzman et al., 1995
pBAD33-AHA4171	pBAD33 with AHA_4171 of ATCC7966^T^, Cm^r^	This work
pBAD33-Fgi-1	pBAD33 with *fgi-1* of AH-3, Cm^r^	This work
pBAD33-M15300	pBAD33 with M001_15300 of HM21, Cm^r^	This work

*Rif^r^, rifampicin resistant; Cm^r^, chloramphenicol resistant; Sp^r^, spectinomycin resistant*.

### General DNA Techniques and Nucleotide Sequencing

DNA manipulations were carried out according to standard procedures (Sambrook et al., 1989). DNA restriction endonucleases were obtained from Promega. T4 DNA ligase and alkaline phosphatase were obtained from Invitrogen and GE Healthcare, respectively. Polymerase chain reaction (PCR) was performed using the BioTaq DNA polymerase (Ecogen) in a Gene Amplifier PCR System 2400 Perkin Elmer Thermal Cycler. Plasmid DNA for sequencing was isolated by Qiagen plasmid purification kit (Qiagen, Inc. Ltd.) as recommended by the suppliers. Double-strand DNA sequencing was performed by using the Sanger dideoxy-chain termination method (Sanger et al., 1977) with the BigDye Terminator v3.1 cycle sequencing kit (Applied Biosystem). Custom-designed primers used for DNA sequencing were purchased from Sigma-Aldrich. The DNA sequences were compared with those available in the GenBank and EMBL databases at the National Center for Biotechnology Information (NCBI) (Altschul et al., 1997).

### Constructions of Defined in Frame Mutants

The in-frame deletion of *fgi* locus and the single defined deletion of AHA_4171 and *fgi-1* were obtained by allelic exchange as described by Milton et al. (1996) using the primers listed in Table 2. Briefly, deletion of *fgi* locus of *A. piscicola* AH-3 was performed by amplification of DNA regions upstream of *fgi-*1 and downstream of *fgi-12* in two sets of asymmetric PCRs. The single defined deletions were performed by amplification of DNA regions upstream and downstream of *fgi-1* of *A. piscicola* AH-3 and AHA_4171 of *A. hydrophila* ATCC7966^T^ in two sets of asymmetric PCRs. Primer pairs A-Flgi1 and B-Flgi1, and C-Flgi1 and D-Flgi1, amplify DNA fragments of 776bp (AB-Fgi1) and 687bp (CD-Fgi1) upstream and downstream of *fgi-1*, respectively. Primer pairs C-Flgi12 and D-Flgi12 amplify a DNA fragment of 732 bp (CD-Fgi12) downstream of *fgi-12*. Primer pairs A-4171 and B-4171, and C-4171 and D-4171, amplify DNA fragments of 610 bp (AB-4171) and 764 bp (CD-4171) upstream and downstream of AHA_4171, respectively. DNA fragments AB-Fgi1 and CD-Fgi1, AB-Fgi1 and CD-Fgi12, or AB-4171 and CD-4171 were annealed at their overlapping regions and amplified as a single fragment using primers A-Fgi1 and D-Fgi1, A-Fgi1 and D-Fgi12, or A-4171 and D-4171, respectively. The AD fusion products were purified, *Bgl*II or *Bam*HI digested, ligated into *BglII*-digested and phosphatase-treated pDM4 vector (Milton et al., 1996) and electroporated into *E. coli* MC1061 (λ*pir*) and plated on chloramphenicol plates at 30°C to obtain pDM-Fgi-1, pDM-AH3Fgi, and pDM-AHA4171 plasmids, respectively. Introduction of the plasmids into *A. piscicola* AH-3 rifampicin-resistant (Rif^r^), AH-405, or *A. hydrophila* ATCC7966-Rif was performed by triparental matings using the *E. coli* MC1061 *(*λ*pir*) containing the insertion constructs and the mobilizing strain HB101/pRK2073. Transconjugants were selected on plates containing chloramphenicol and rifampicin. PCR analysis confirmed that the vector had integrated correctly into the chromosomal DNA. After sucrose treatment, transformants that were rifampicin-resistant (Rif^r^) and chloramphenicol sensitive (Cm^S^) were chosen and confirmed by PCR.

**Table 2:** Primers used for the construction of the in frame defined mutants.

**Primer name**	**Sequence in 5^**′**^ to 3^**′**^ direction**
***A. piscicola*** **AH-3**	
A-Flgi1	CGC$\underline{\underline{\text{GGATCC}}}$GACTGTACCCGTTTCAATCA
B-Flgi1	CCCATCCACTAAACTTAAACAGATCACCTCGAACTCGAAA
C-Flgi1	TGTTTAAGTTTAGTGGATGGGGTGCAACAACTGTTTGGAG
D-Flgi1	CGC$\underline{\underline{\text{GGATCC}}}$AGAGCCTGACCTCAATCAA
C-Flgi12	TGTTTAAGTTTAGTGGATGGGGGAACCTTAAATGCCATGA
D-Flgi12	CGC$\underline{\underline{\text{GGATCCC}}}$AGTCTTCAGCTTCCATCC
***A. hydrophila*** **ATCC7966**^**T**^	
A-4171	CGC*AGATCT*CGATGGAACGACTGATCCAC
B-4171	CCCATCCACTAAACTTAAACAGGGCACCAATACGCTGACTT
C-4171	TGTTTAGTTTAGTGGATGGGGAAAAAGGGGAAAAGCCATC
D-4171	CGC*AGATCT*GTAAGCGCAATGCTTGTTCA

*Overlapping regions in primers B and C are underlined*.

*BglII site is in italic letters and BamHI site is double-underlined in primers A and D*.

### Plasmid Construction

Plasmid pBAD33-AHA4171, pBAD33-Fgi-1 and pBAD33-M15300, containing the complete AHA_4171 gene from *A. hydrophila* ATCC7966^T^, *fgi-*1 gene from *A. piscicola* AH-3 and M001_15300 gene from *A. veronii* HM21, respectively, under the arabinose promoter (p_BAD_) on pBAD33 (Guzman et al., 1995) were obtained by PCR amplification of genomic DNAs. Oligonucleotides 5′-GCG*CCCGGG*AAATCCAGCAGCTTCAATGG-3′ and 5′-GCGTCTAGATGT ATTAGG GCCGCTAGGTG-3′ generated a band of 1204 bp containing the AHA_4171 gene. Oligonucleotides 5′-GGC*GATATC*GGTAGCCTTGCCCATTTTCT-3′ and 5′-GGCTC TAGAGCGA CAGGTAATCCCACACT-3′ generated a band of ~1,250 bp containing the *fgi-*1 or M001_15300 gene (the *Sma*I site is underlined, the *Xba*I site double-underlined and the *EcoR*V site is in italic). The amplified band containing the AHA_4171 gen was *Sma*I and *Xba*I digested and the amplified bands containing the *fgi-*1 or M001_15300 gene digested with *EcoR*V and *Xba*I. The digested bands were independently ligated into *Sma*I-*Xba*I digested pBAD33 vector to construct the pBAD33-AHA4171, pBAD33-Fgi-1 and pBAD33-M15300 plasmids. Recombinant plasmids were introduced by electroporation into the *E. coli* DH5α (Hanahan, 1983) and sequenced. For complementation assay, the recombinant plasmids were introduced into the AH-3ΔFgi*-*1 or ATCCΔAHA4171 mutants (Rif^r^) by triparental mating using the *E. coli* DH5α containing the recombinant plasmids and the mobilizing strain HB101/pRK2073. Transconjugants were selected on plates containing chloramphenicol and rifampicin.

### Motility Assays

Fresh bacterial grown colonies were transferred with a sterile toothpick onto the center of a soft agar plate (1% tryptone, 0.5% NaCl, 0.25% agar). Plates were incubated face up for 24-48 h at 25°C and motility was assessed by examining the migration of bacteria through the agar from the center toward the periphery of the plate. Moreover, swimming motility was assessed by light microscopy observations in liquid media.

### *Aeromonas* Polar Flagella Purification

Purification of *Aeromonas* polar flagella was carried out from overnight cultures in tryptic soy broth (TSB) at 25°C. Cells were collected by centrifugation at 5,000 × g, and suspended in 100 mM Tris buffer (pH 7.8). Flagella were removed from the cells by shearing in a vortex with a glass bar for 3–4 min, and then passing repetitively (minimum six times) through a syringe. Cells were removed by centrifugation at 8,000 × g for 30 min, and the supernatant centrifuged at 18,000 × g for 20 min. From the remaining supernatant the flagella were pelleted by ultracentrifugation at 100 000 x *g* for 60 min, and resuspended in 100 mM Tris (pH 7.8) plus 2 mM EDTA buffer. This flagella enriched fraction was purified using a cesium chloride gradient by ultracentifugation at 60 000 × g for 48 h. The band containing the flagella was collected, the cesium chloride removed by extensive dialysis against the same buffer (100 mM Tris 2 mM EDTA). Purified flagella were analyzed by SDS-PAGE or by mass spectrometry analysis.

### Mass Spectrometry Analysis of Flagellin Glycopeptides

Purified flagellin was proteolytically digested using trypsin diluted in 50 mM ammonium bicarbonate at a 30:1 protein to enzyme ratio overnight at 37°C, as described previously (Twine et al., 2008). Flagellin digests were analyzed by reversed phase nano liquid chromatography tandem mass spectrometry (nLC-MS/MS) using an M-class high performance liquid chromatography (HPLC) system (Waters Corp). Peptides were first loaded onto a 5 mm × 300 μm C8 (Dionex) and a 20 mm × 180 μm C18 (Waters Corp) trap columns in series. They were subsequently eluted onto a 100 mm × 100 μm C18 BEH column (Waters Corp) for analytical separation. The gradient was applied at 0.5 μL/min as follows: 1–45% solvent B over 18 min, 45–85% solvent B over 3 min, 85–1% solvent B of 1 min, and finally an 8 min re-equilibration at 1% solvent B. Solvent A was 0.1% formic acid in HPLC grade water (Fisher Scientific) and solvent B was 0.1% formic acid in HPLC grade acetonitrile. Peptides were analyzed by electrospray ionization (ESI) using a Synapt G2 quadrupole time of flight (QTOF) mass spectrometer (Waters Corp). Flagellin glycopeptide spectra were annotated by *de novo* sequencing.

### Immunological Methods

Western blot of purified polar flagella was performed as briefly described (Merino et al., 2014). After SDS-PAGE separation of flagella and transfer to nitrocellulose membrane at 1.3 A for 1 h, the membranes were blocked with bovine serum albumin (3 mg/mL), and probed with polyclonal rabbit anti-AH-3 polar flagella antibodies (1:1,000) (Gavin et al., 2002). The unbound antibody was removed by three washes in phosphate buffered saline (PBS), and a goat anti-rabbit immunoglobulin G alkaline phosphatase conjugated secondary antibody (Sigma) (1:1,000) was added. The unbound secondary antibody was removed by three washes in PBS. The bound conjugate was then detected by the addition of 5-bromo-4-chloroindolylphosphate disodium-nitroblue tetrazolium. Incubations were carried out for 1 h, and washing steps with 0.05% Tween 20 in PBS were included after each incubation step.

## Results

### Identification of the Polar Flagellin Glycosylation Islands (*fgi*) in *A. piscicola* AH-3

**Figure 2:**
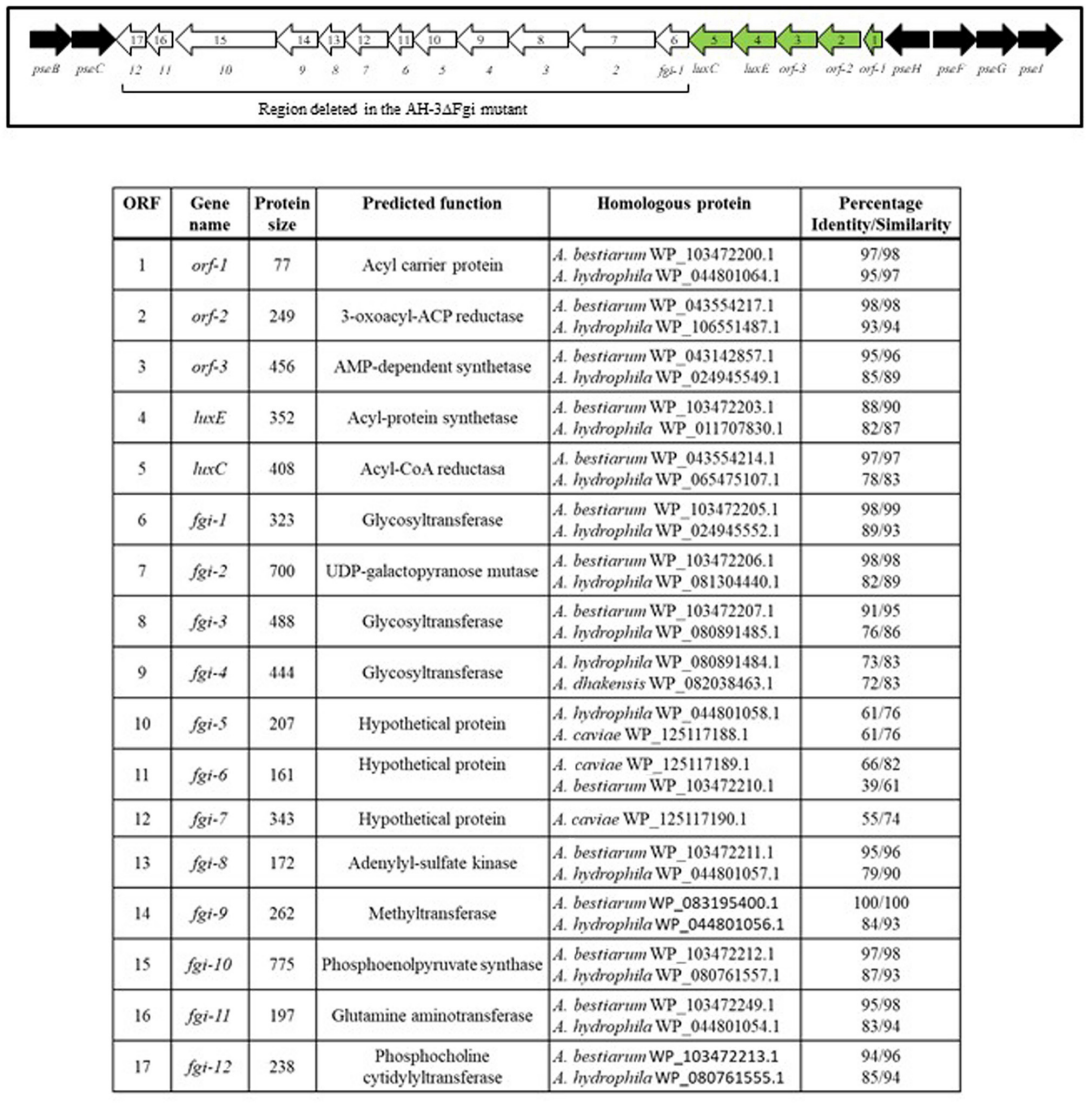
Schematic representation of *A. piscicola* AH-3 polar flagellum glycosylation island (FGI). Pseudaminic acid biosynthetic genes, in black; polar flagellum glycosylation genes that were deleted in the AH-3ΔFgi mutant, in white; and genes involved in the channeling of fatty acids into the fatty aldehyde substrate, in green.

**Figure 3:**
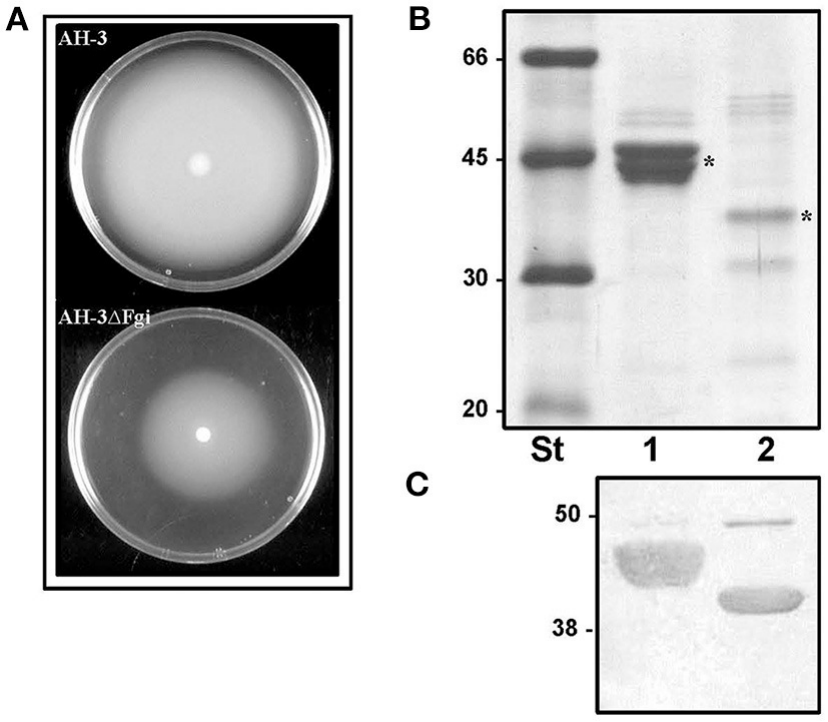
**(A)** Motility of *A. piscicola* AH-3 and AH-3ΔFgi mutant grown for 20 h at 25°C on soft agar. Purified polar flagellum from AH-3 (lane1) and AH-3ΔFgi mutant (lane 2) were analyzed using 12% SDS-PAGE **(B)** and by western blot using *A. piscicola* anti-AH-3 polar flagella antiserum (1:1,000) **(C)**. Size standard (St). Polar flagellins (*).

In *A. piscicola* AH-3 and *A. hydrophila* ATCC7699^T^ the genes of the pseudaminic acid biosynthesis locus, *pseB* (*flmA*), *pseC* (*flmB*), *pseF* (*neuA*), *pseG* (*flmD*), *pseI* (*neuB*) and *pseH* (*flmH*), are distributed in two different chromosomal locations. The *pseBC* genes are separated from the *pseHFGI* genes by 18 Kb in *A. piscicola* AH-3, and by 27 Kb in *A. hydrophila* ATCC7699^T^ (Figure 1). The *A. piscicola* AH-3 fragment contains 17 open reading frames (ORFs) transcribed in the same direction. The five ORFs downstream of *pseH* (orf1-5) encode proteins that have shared homology to proteins involved in the channeling of fatty acids into the fatty aldehyde substrate. The remaining ORFs, named *fgi-1* to *fgi-12*, encode proteins with shared homology to a variety of transferases, many of them glycosyltransferases (Figure 2). In order to establish whether genes within this fragment are involved in the biosynthesis of the heptasaccharide that modifies the polar flagellin of *A. piscicola* AH-3, an in frame deletion of all the genes between the gene downstream of *pseC* and the gene downstream of the acyl-CoA reductase (*luxC*) was generated. This mutant, denoted AH-3ΔFgi in *A. piscicola* AH-3, showed reduced swimming motility in liquid medium and by light microscopy, in comparison to the wild-type strain. Furthermore, its motility in soft agar showed a decreased radial expansion (34% reduction) in relation with the wild-type strain (Figure 3A). Analysis of purified polar flagellum, by SDS-PAGE and western blot, using anti-AH-3 polar flagellum antibodies (Figures 3B,C), showed polar flagellins with lower molecular weight in the AH-3ΔFgi mutant than in the wild-type strain. This difference fit with the loss of most part of the heptasaccharide. Furthermore, the amounts of polar flagellins assembled on the bacterial surface of the AH-3ΔFgi mutant are 3.5 times lower than in the wild-type AH-3 (Figure 3B).

### Bioinformatic Characterization of the Polar Flagellin Glycosylation Islands (FGIs) in *Aeromonas*

After identifying and demonstrating the role of this FGI in the polar flagellin glycosylation of *A. piscicola* AH-3, we characterized the FGIs in 265 mesophilic *Aeromonas* genomes retrieved from Genbank. The *A. piscicola* AH-3 PseC (an aminotransferase which aminates at C4 the UDP-4-keto-6-deoxy-L-AltNAc) and PseI (a pseudaminic acid synthase which pyruvylates the 2,4,6-tridoxy-2,4-NAc-L-altrose) were used to locate the pseudaminic acid biosynthesis clusters. Homologous *pseC* and *pseI* genes were found in 50 of 52 complete genomes and were not detected in the *Aeromonas media* WS and the *Aeromonas veronii* AVNIH1 genomes. Homologs to both genes were also detected in 202 of 213 non-complete genomes. However, 42 of these 202 non-complete genomes present assembly breaks inside this region and these genes are in different contig or scaffold and therefore were not be used in this study.

In 194 of 210 genomes retained for analysis (50 complete and 160 non-complete genomes) the genes related to polar flagellin glycosylation were found aggregated in a genomic island delimited by the pseudaminic acid biosynthetic genes *pseB* and *pseI*. However, in 16 of 160 non-complete genomes, the genomic island was delimited by the pseudaminic acid biosynthetic genes *pseB* and *pseF*. Homologs of the pseudaminic acid biosynthesis genes *pseBCFI* were present in all of polar flagellin genomic island of the strains analyzed. However, the presence of *pseG* and *pseH* homologs was variable. While 125 strains have both genes, 58 have only the *pseG*, 22 have only the *pseH* and four strains do not possess either *pseG* or *pseH*.

**Figure 4:**
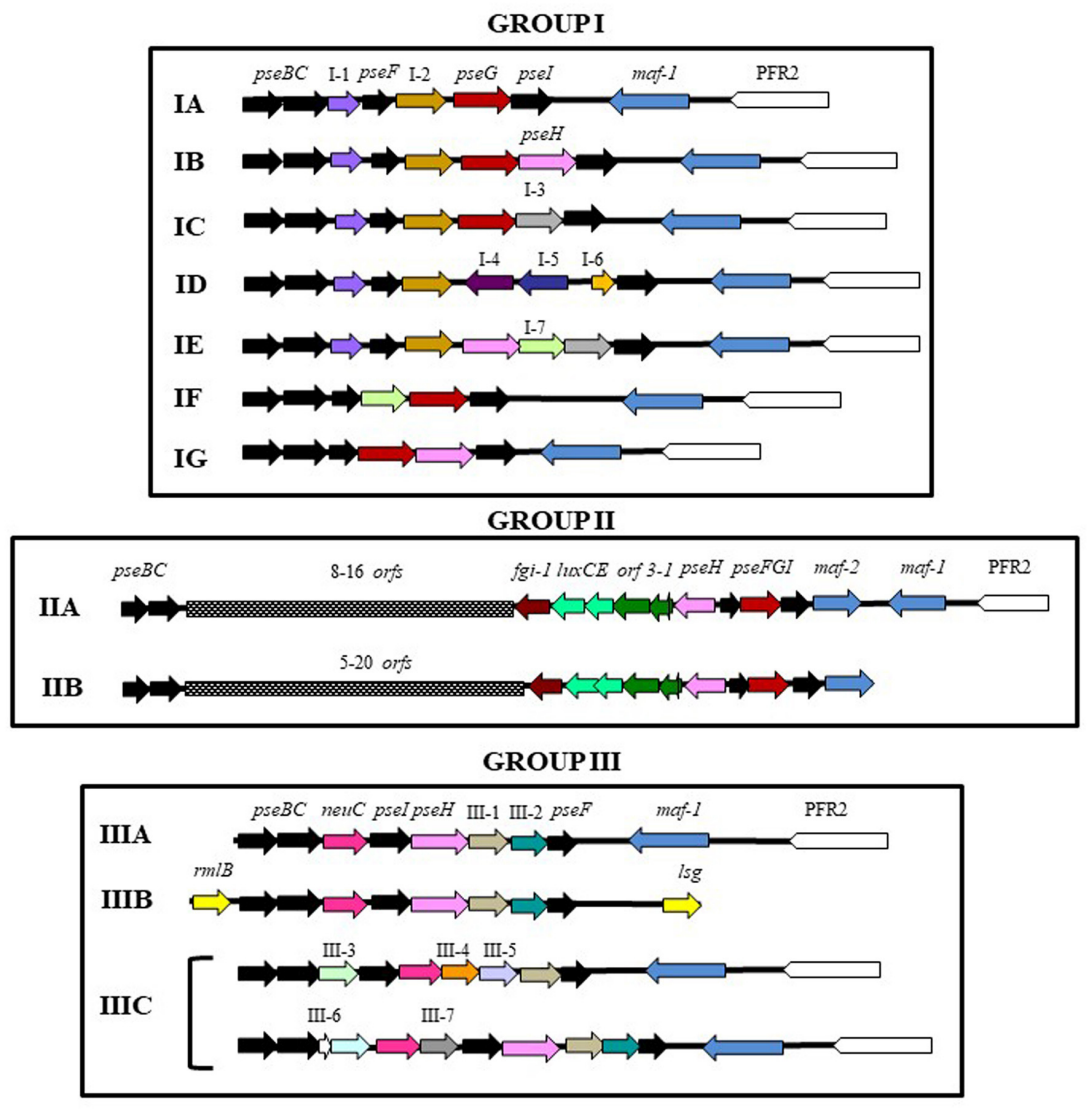
Schematic diagram of the polar flagellin glycosylation islands (FGIs) of mesophilic *Aeromonas*. PFR2: Polar flagellum region 2. Black, red and pink arrows shows pseudaminic biosynthetic genes. In group I, arrows labeled as I-1 (violet): alpha/keto reductase; I-2 (brown): class III aminotransferase; I-3 (gray): glucuronic acid dehydrogenase; I-4 (purple): HAD-IA family hydrolase; I-5 (dark-blue): methyltransferase; I-6 (orange): hypothetical protein; I-7 (green): deacetylase. In group II, *orf1*: acyl carrier protein; *orf2*: 3-oxoacyl-ACP-reductase; *orf3*: AMP-dependent synthetase; and *fgi-1*: glycosyltransferase. In group III, yellow arrows (*rmlB, lsg*) shows O-antigen biosynthetic genes, and arrows labeled as III-1 (light-brown): CBS-domain containing protein; III-2 (blue-green):Gfo/Idh/MocA family oxidoreductase; III-3 (light-green): GNAT family N-acetyltransferase; III-4 (dark-orange): pyridoxal phosphate-dependent transferase; III-5 (light-violet): methyltransferase; III-6 (light-blue): acyl dehydratase; and III-7 (dark-gray): pyridoxal phosphate-dependent transferase.

The genomic regions were observed to be complex, with low average %GC of 49.4–55% and a length ranging from 7.5 to 32.8 Kb. Based on their structure and presence of gene homologs, this genomic glycosylation island was categorized into three main distinct groups across *Aeromonadaceae* (Figure 4 and Supplementary Table 1).

Group I shows a small polar flagellin glycosylation genomic island ranging from 7.5 to 9.5 Kb, downstream of polar flagellum region 2 (Wilhelms et al., 2011), containing the pseudaminic acid biosynthesis genes, *pseBCFI*, and genes involved in transference or modification of functional groups, such as deacetylases, reductases, methyltransferases and aminotransferases. In this region, only a gene encoding a glycosyltransferase orthologous to the Maf-1 motility accessory factor of *A. caviae* Sch3N was found (Parker et al., 2014). This gene is between the polar flagellum region 2 and the flagellin glycosylation island (FGI). Group I contains 96 strains of mesophilic *Aeromonas* and was divided in seven subgroups (A-G). Subgroup IA shows only a gene encoding an alpha/keto reductase between *pseC* and *pseF*, and a gene encoding a class III aminotransferase between *pseF* and *pseG*. This subgroup contains 39 strains: 27 belong to *A. hydrophila*, four belong to each of the *A. allosaccharophila* and *A. veronii* species, three belong to *Aeromonas* sp. and one belongs to *A. cavernicola*. Since the genomic region containing the polar flagella region 2 and the FGI of *A. cavernicola* are in different genomic contigs, it is impossible to know if they are adjacent. Subgroup IB and IC show identical gene distribution as IA, but they have additional genes between *pseG* and *pseI*. Subgroup IB shows a gene which encoded an *N*-acetyltransferase (PseH) and IC has a gene encoding a glucuronic acid dehydrogenase. Subgroup IB contains 24 strains: 10 strains belong to the *A. caviae* species, three belong to *Aeromonas* sp., *two* belong to each of the *A. encheleia* and *A. hydrophila* species, and one belongs to each *A. aquatic, A. eucrenophila, A. lusitana, A. media, A. molluscorum, A. rivipollensis*, and *A. tecta* species. Subgroup IC contains 17 strains: seven belong to *A. veronii*, three belong to each of the *A. bivalvium* and *A. caviae* species, and one belongs to each *A. jandaei, A. sobria, Aeromonas* sp. and *A. veronii bv sobria* species. Subgroup ID and IE show identical gene distribution as subgroup IA in the fragment contained between *pseB* and the gene encoding the class III aminotransferase. None of them shows a gene encoding an ortholog of PseG. Subgroup ID has three genes between the class III aminotransferase gene and *pseI*: a hypothetical protein, a gene encoding a methyltransferase and a gene encoding a protein belonging to the HAD-IA family hydrolases. This subgroup includes four strains belonging to each of the *A. jandaei, A. sobria, Aeromonas* sp. and *A. veronii* species. In *A. sobria* 08005 and *A. veronii* FC951 this region also contains a transposase gene. Subgroup IE shows a gene encoding a protein orthologous to PseH downstream of the class III aminotransferase gene and two genes between *pseH* and *pseI*: one encoding a deacetylase and the other, encoding a glucuronic acid dehydrogenase. This subgroup includes six strains within the *A. caviae* and *A. veronii* species (four and two strains, respectively). Subgroups IF and IG do not show homologous to the alpha/keto reductase and class III aminotransferase genes, which are present in the other subgroups. In subgroup IG, genes involved in the pseudaminic acid biosynthesis, *pseBCFGHI*, are contiguous and in subgroup IF only a gene encoding a deacetylase is located between *pseF* and *pseG*. Subgroup IF includes one strain in each of the *A. caviae* and *A. veronii bv. sobria* species and IG includes two strains of *A. media*, and one strain in each of the *A. caviae* and *A. rivuliii* species. However, in the *A. caviae* GEO_23_Down_B, the genomic region containing the polar flagella region 2 and the FGI are not adjacent (Figure 4 and Supplementary Table 1).

The second FGI group (Group II) was found in 98 strains of mesophilic *Aeromonas* and the FGI is more complex than in group I. Pseudaminic acid biosynthesis genes, *pseBC* and *pseFGHI*, are clustered in two genomic regions. These regions are separated by a fragment of variable length which encodes a variable number of transferases: these are mainly glycosyltransferases, but there are also methyl-, acetyl-, and phospho- transferases depending on the strain. There are also several proteins involved in flatty acid synthesis: an acyl carrier protein (*orf1*), a 3-oxoacyl-ACP-reductase (*orf2*), an AMP-dependent synthetase (*orf3*), an acyl-protein synthetase (*luxE*), and an acyl-CoA reductase (*luxC*). A high number of transposases were also found in this region of 32 strains. All the strains, except *A. caviae* Aer268, show a conserved and specific uncharacterized glycosyltransferase downstream of *luxC*, that we named flagella glycosylation island 1, *fgi-1*. Furthermore, they also show, downstream of *pseI*, a gene encoding a motility factor protein orthologous to Maf-2 of *A. piscicola* AH-3 (Canals et al., 2007). According to the chromosomal localization of FGI we differentiate two subgroups: IIA and IIB. In subgroup IIA, the FGI is downstream of polar flagellum region 2; however, this is not the location in subgroup IIB, although in all *A. veronii* and *A. caviae* strains, as well as in 1 of *A. jandaei* and 2 of *Aeromonas* sp. strains it is only 4.5–5.5 Kb away. Independently of their chromosomal localization, all strains have a gene encoding a motility factor protein orthologous to Maf-1 downstream of polar flagella region 2 (Parker et al., 2014). Subgroup IIA contain 32 mesophilic *Aeromonas* strains: 19 of these strains belong to *A. veronii*, five belong to *A. caviae*, two belong to each of the *A. enteropelogenes, A. piscicola* and *A. schubertii* species, and 1 belongs to *A. bestiarum* and *Aeromonas* sp. Subgroup IIB include 66 strains: 30 belong to *A. hydrophila*, 15 to *A. dhakensis*, 9 to *A. veronii*, 5 to *Aeromonas* sp., 2 to *A. caviae* and *A. jandaei*, and 1 to *A. bestiarum, A. enteropelogenes* and *A. hydrophila* subsp*. ranae*.

The third FGI group (Group III) was found in 16 strains of mesophilic *Aeromonas* that shows a small FGI delimited by the pseudaminic acid biosynthetic genes *pseB* and *pseF* instead of *pseI*. Furthermore, it also contains a gene encoding an ortholog of UDP-N-acetylglucosamine 2-epimerase, *neuC*, related to the sialic acid biosynthesis. The group was divided in three subgroups (A-C) according to their chromosomal location, distribution of pseudaminic acid biosynthetic genes and additional genes. Subgroup IIIA and IIIB shows *neuC* between *pseBC* and *pseIH*, as well as two additional genes between *pseH* and *pseF*. These additional genes encode a CBS-domain containing protein or an alcohol dehydrogenase and a Gfo/Idh/MocA family oxidoreductase. These two subgroups differ in the chromosomal location of the FGI. In IIIA, it is adjacent to *maf-1* which is downstream of the polar flagella region 2; however, genes that could be involved in the biosynthesis of O-antigen lipopolysaccharide are found upstream and downstream of the IIIB glycolyslation island. Subgroup IIIA contain four strains belonging to species *A. austraiensis, A. caviae, A. veronii* and *Aeromonas* sp., although in *A. caviae* CH129, the genes of GGI are transcribed in the same direction than genes of polar flagella region 2. Subgroup IIIB contain eight strains: six belong to *A. veronii* and the other to *A. caviae* and *A. simiae*. Furthermore, *A. caviae* 8LM and *Aeromonas* sp. ASNIH5 which shows subgroup IB, also have subgroup IIIB (Figure 4 and Supplementary Table 1). Subgroup IIIC only contains four strains: one belongs to each of *A. media* and *Aeromonas* sp., and two belong to *A. hydrophila*. The chromosomal location of this GGI is such as IIIA but has additional genes. *A. media* S2_009_000_R3_19 show a gene which encodes a GlcNAc-PI de-*N*-acetylase, between *pseI* and *neuC*, and two additional genes which encode a GNAT family *N*-acetyltransferase and an acyl dehydratase, between *neuC* and *pseC*. The other strains do not show a gene orthologous to *pseH* and *pseI* is located upstream *neuC*. Furthermore, they show an additional gene downstream of *pseC* which encodes a GNAT family *N*-acetyltransferase; however, they have three additional genes upstream of *pseF* which encode for a CBS-domain containing protein, a methyltransferase and a pyridoxal phosphate-dependent transferase (Figure 4 and Supplementary Table 1).

### Bioinformatic Characterization of Glycosyltransferase Fgi-1

With the exception of *A. caviae* Aer268, all mesophilic *Aeromonas* strains grouped within FGI Group II (97 strains), showed a conserved glycosyltransferase downstream of *luxC*. In *A. piscicola* AH-3, this glycosyltransferase was named flagella glycosylation island 1, *fgi-1* (Figure 4). The Fgi-1 protein sequence has 327 amino acid residues, a predicted molecular weight of 37.44 kDa and a predicted pI of 4.98. Homologous proteins in polar FGI group II range between 313-327 residues, with predicted molecular weights of 35.67-37.39 kDa and predicted isoelectric points (pI) of 4.98-6.16 (Supplementary Figure 1). Sequence identity ranges between 100 and 54% across all sequences and according to CDD and SMART, all sequences shows a glycosyltransferase family 2 (GT-2) conserved domain in the first 100 amino acids. No signal peptide or transmembrane domains were predicted in the protein sequences according to SignalP-HMM v4.1 and TMHMM Server v2.0, therefore Fgi-1 is predicted to be active in the bacterial cytoplasm.

**Figure 5:**
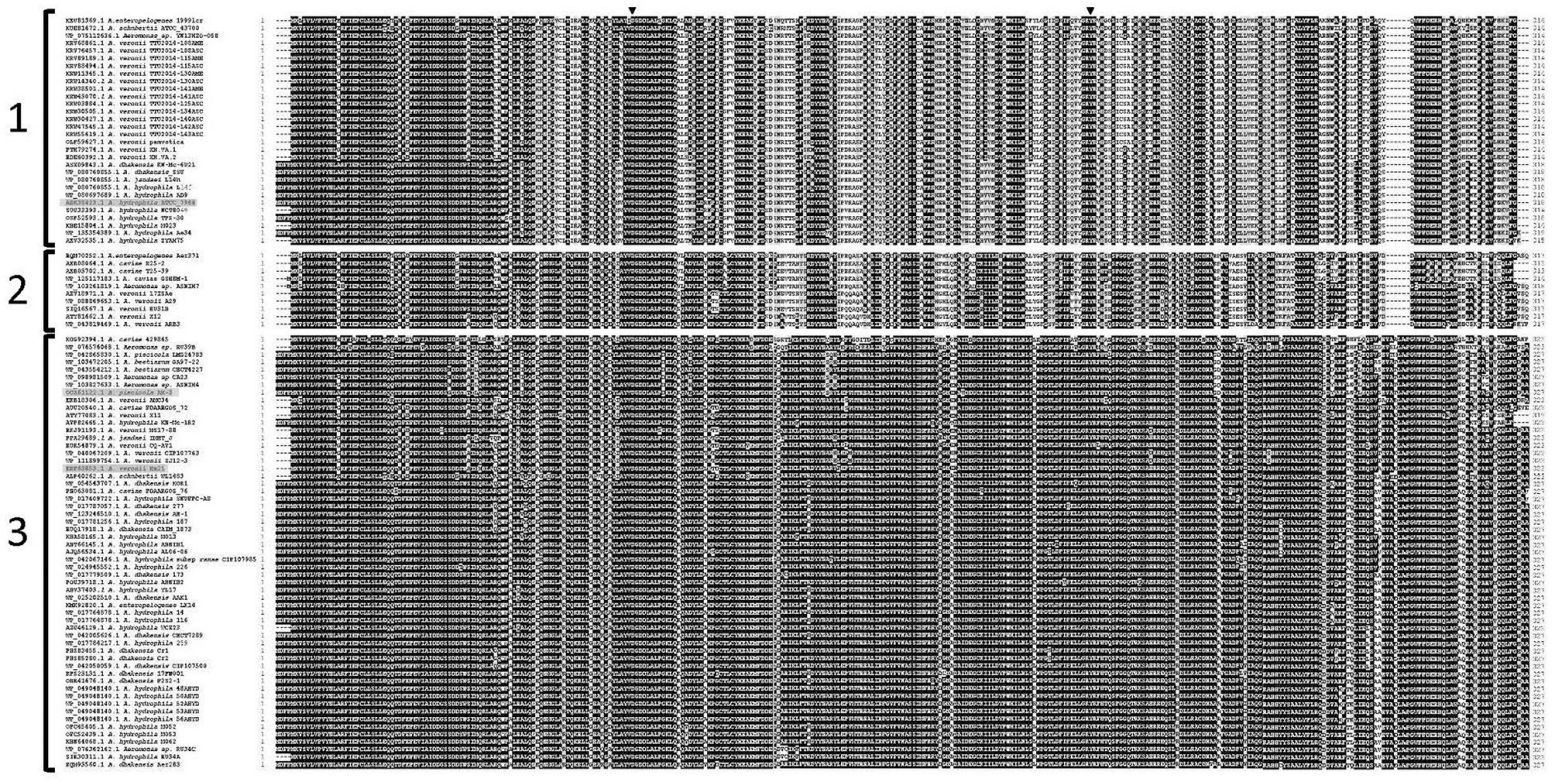
Schematic alignment, performed by ClustalW, of the Fgi-1 amino acid sequences of mesophilic *Aeromonas* strains with polar flagella glycosylation islands group II. Fgi-1 groups are separated by brackets and sequences labeled in gray were used in the complementation assays. Black triangles show the Asp94 (D94) and Tyr215 (Y215) interacting residues at the protein pocket.

**Figure 6:**
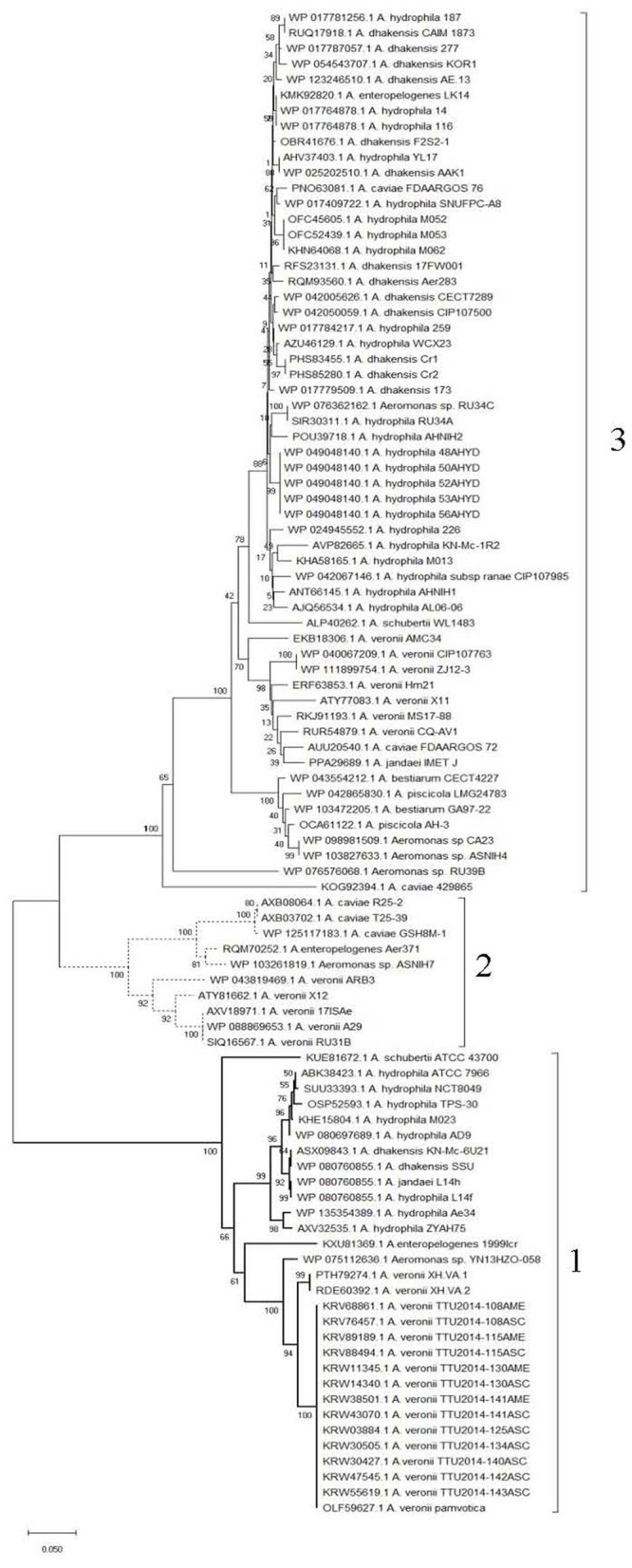
Phylogenetic tree generated by the neighbor-joining method on the basis of the Fgi-1 amino acid sequences. Brackets show the three Fgi-1 groups.

Clustering of the 97 Fgi-1 protein sequences shows higher conservation in the N-terminal sequence containing the GT-2 domain compared to the rest of the sequence. This highlighted three different groups with more than 80% intragroup sequence identity (Figure 5 and Supplementary Figure 1). A phylogenetic tree generated by the neighbor-joining method on the basis of the Fgi-1 amino acid sequences also shows these three groups (Figure 6). The first group (Group 1) contains 30 mesophilic *Aeromonas* strains: 16 belong to *A. veronii*, eight belong to *A. hydrophila, two* belong to *A. dhakensis*, one belongs to each of the *A. jandaei, A. shubertii, A. enteropelogenes* and *Aeromonas* sp. The second group (Group 2) contains 10 mesophilic *Aeromonas* strains: five belong to *A. veronii*, three belong to *A. caviae*, and one belongs to each of the *A. enteropelogenes* and *Aeromonas* sp species. The third group (Group 3) is the most extended and contain 57 mesophilic *Aeromonas* strains: 22 belong to *A. hydrophila*, 13 belong to *A. dhakensis*, seven belong to *A. veronii*, four belong to *Aeromonas* sp, three belong to *A. caviae*, two belong to each of the *A. piscicola* and *A. bestiarum* species, and one belongs to each of the *A. jandaei, A. shubertii, A. enteropelogenes* and *A. hydrophila* subs *ranae*.

The Fgi-1 of *A. piscicola* AH-3 (OCA61122.1), C7U63_02395 of *A. veronii* 17ISAe (AXV18971.1) and AHA_4171of *A. hydrophila* ATCC7966 (ABK38423.1) were used as models of group 1, 2, and 3, respectively, to analyze intergroup sequence identities. Sequence identities were 66, 60, and 55% between groups 2 and 3, 1 and 2, and 1 and 3, respectively.

**Figure 7:**
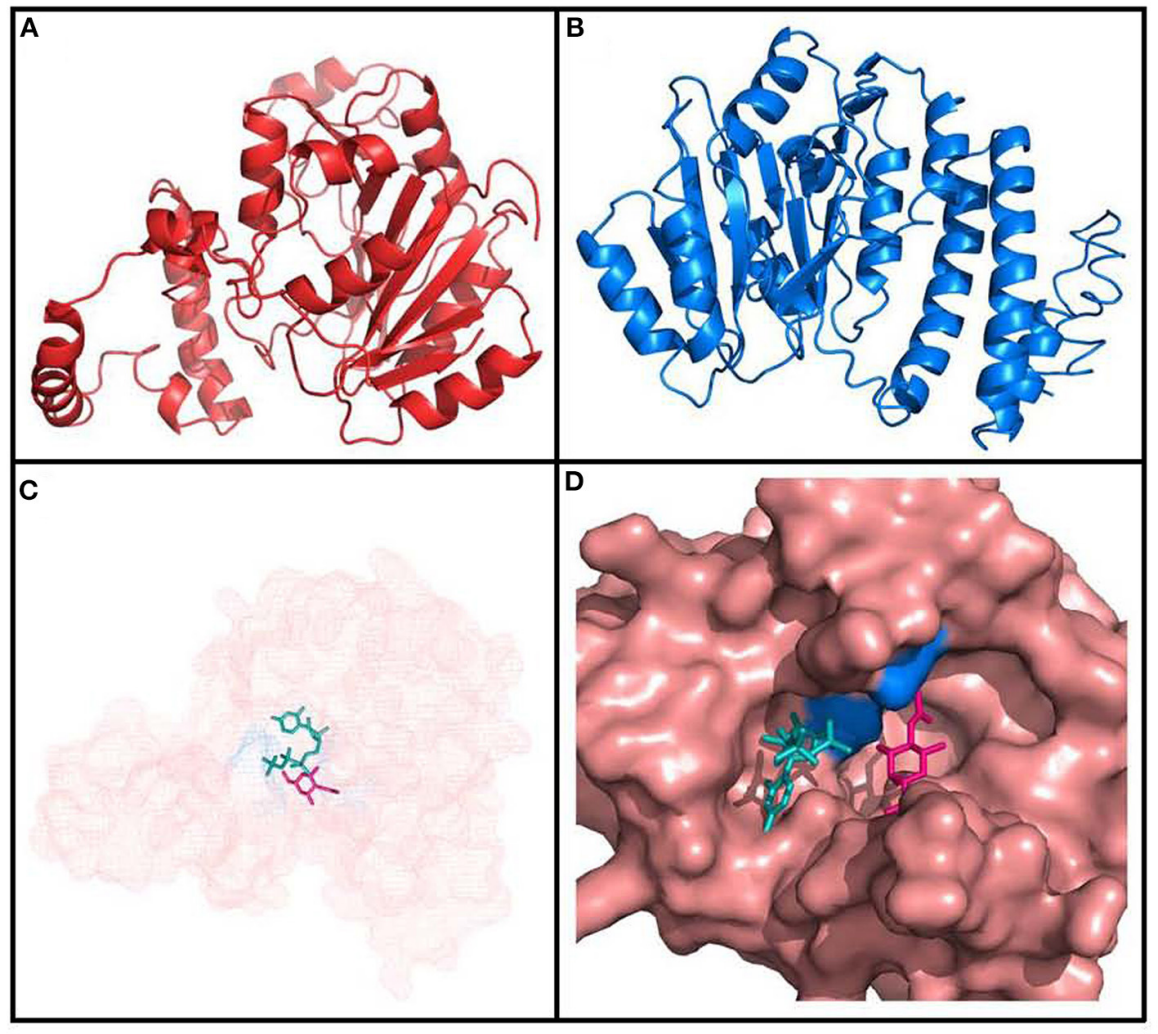
Tertiary structure of Fgi-1 as modeled by **(A)** Phyre2, and **(B)** I-Tasser. **(C)** The glycosyltransferase main pocket [amplified in **(D)**] has predicted binding sites with complex carbohydrates, as *N*-acetyl-*D*-hexosamine (pink), energy donors (ATP/CTP, in green), and Mg ions. The ASP94 and TYR215 aminoacids (blue) are predicted to be ligand binding sites.

Secondary structure of the protein analyzed with SOPMA predicted that it is predominantly alpha helixical (41.90%) and random coils (34.56%), with a minor part of the structure predicted to be extended strands (15.60%). Finally, we studied the Fgi-1 tertiary structure using two different methods: homology modeling with Phyre2, and threading modeling with LOMETS in I-Tasser (Figures 7A,B). Both approaches modeled most of the Fgi-1 tertiary structure based on the conformation of available glycosyltransferase enzymes. Phyre2 used five templates (PDB: c2z86D, c5tz8C, c5heaA, d1gg8a and c4hg6A) with >99% confidence and sequence identity of 21 to 18%, while the I-Tasser model obtained a maximum C-Score of−0.6 based on a Family 2 glycosyltransferase from *S. parasanguinis* (PDB: c5heaA).

The Phyre2 model was further sent to the 3DLigandSite server to predict three-dimensional ligand binding sites with crystallized components available on PDB. 3DLigandSite found four possible ligand binding sites with carbohydrates with an average MAMMOTH score of 18.0995 (>7 is significant). In some instances, other possible binding sites with Mg and Cu ions as cofactors were also reported. Sucrose, Galactose, N-acetyl-D-galactosamine, ATP/CTP, ADP/CDP, and Mg were identified as possible heterogens interacting in the most significant protein pocket, and Asp94 and Tyr215 as the active residues interacting with them (Figures 7C,D). The other possible binding sites also predicted interaction with the heterogens N-acetyl-D-galactosamine, galactose, and Mg. These results are strengthened by the total conservation of the aforementioned putative active binding site residues across all homologous Fgi-1 sequences (Figure 5 and Supplementary Figure 1). I-Tasser results largely agreed with the previous results, predicting ligand binding sites with UDP, N-acetyl-D-galactosamine, and alpha-D-mannose, and marking the ASP94 as predicted active site residue.

### Functional Characterization of Fgi-1 of *A. piscicola* AH-3 and AHA_4171 of *A. hydrophila* ATCC7966^T^

**Figure 8:**
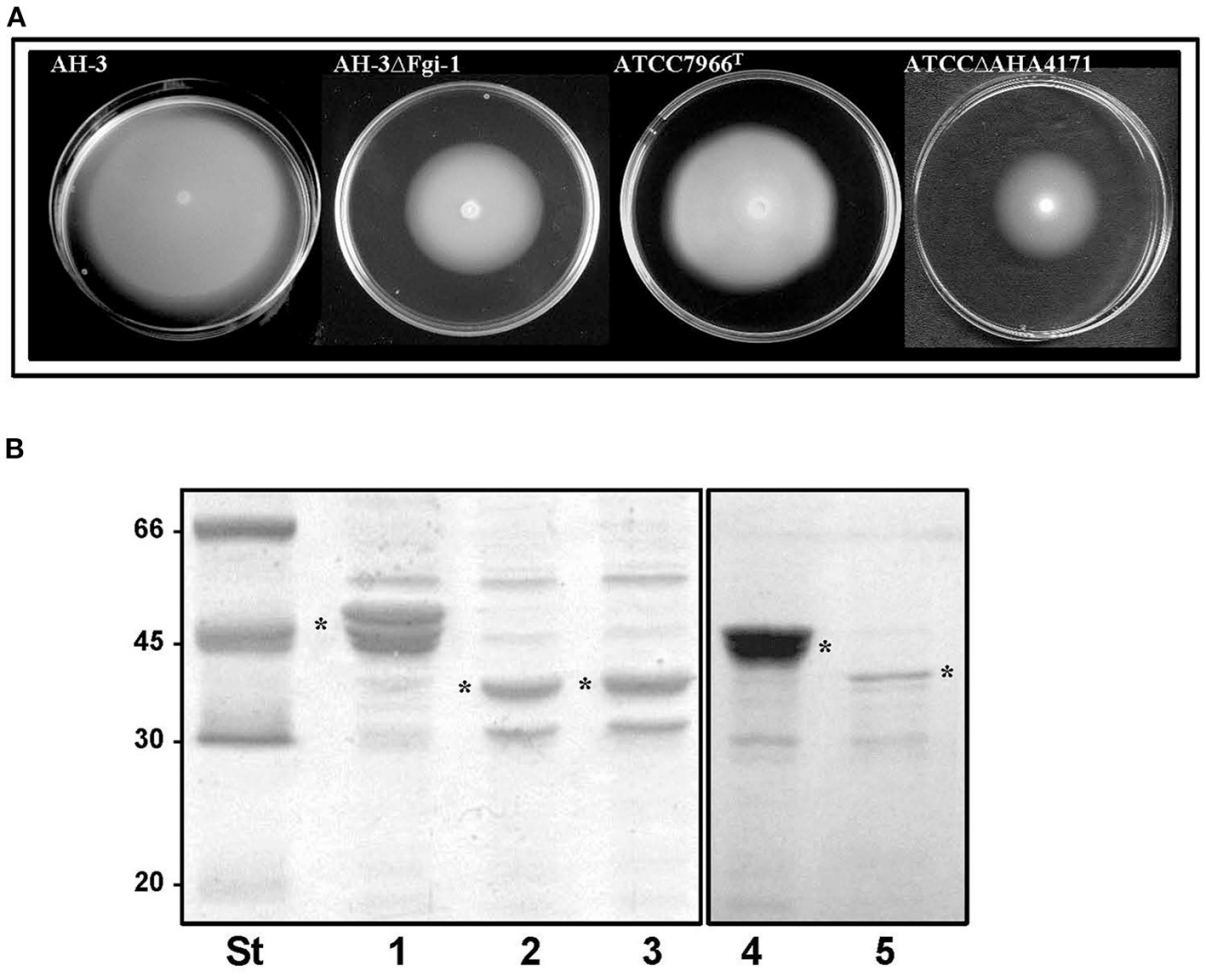
**(A)** Motility on soft agar of *A. piscicola* AH-3 and *A. hydrophila* ATCC7966^T^, and their mutants AH-3ΔFgi-1 and ATCCΔAHA4171grown 20 h at 25°C on soft agar. **(B)** Purified polar flagellum from AH-3 (lane1), AH-3ΔFgi-1 (lane 2), AH-3ΔFgi (lane 3), ATCC7966^T^ (lane 4) and ATCCΔAHA4171 (lane 5) analyzed in 12% SDS-PAGE. Size standard (St). Polar flagellins (*).

Amino acid sequence analysis shows that Fgi-1 of *A. piscicola* AH-3 (OCA61122.1) and AHA_4171 of *A. hydrophila* ATCC7966^T^ (ABK38423.1) are included in different Fgi-1 groups (Figures 5, 6). While Fgi-1 of *A. piscicola* AH-3 belongs to group 1, AHA_4171 of *A. hydrophila* ATCC7966^T^ belongs to group 3. To elucidate their involvement in polar flagellum glycosylation, we constructed specific in-frame mutants in the Fgi-1 of *A. piscicola* AH-3 (AH-3ΔFgi*-*1) and the AHA_4171 of *A. hydrophila* ATCC7966^T^ (ATCCΔAHA4171) using the suicide plasmids pDM-Fgi-1 or pDM-AHA4171, respectively. Specific deletion of genes was confirmed by PCR and sequence analysis. Both mutants showed a reduced swimming motility in liquid medium, by light microscopy, and their motilities in soft agar showed a decreased radial expansion (36 and 39% reduction) in relation with their respective wild-type strain (Figure 8A). Furthermore, as described in the AH-3ΔFgi mutant, analysis of purified polar flagellum, in 12% SDS-PAGE, showed a reduction in the amount of polar flagellins assembled in the bacterial surface and polar flagellins with lower molecular weight, in relation to their respective wild-type strain. In addition, polar flagellins of the AH-3ΔFgi*-*1 mutants show the same molecular weight than the AH-3ΔFgi mutant (Figure 8B).

### Mass Spectrometry Characterization of Fgi-1 of *A. piscicola* AH-3

**Figure 9:**
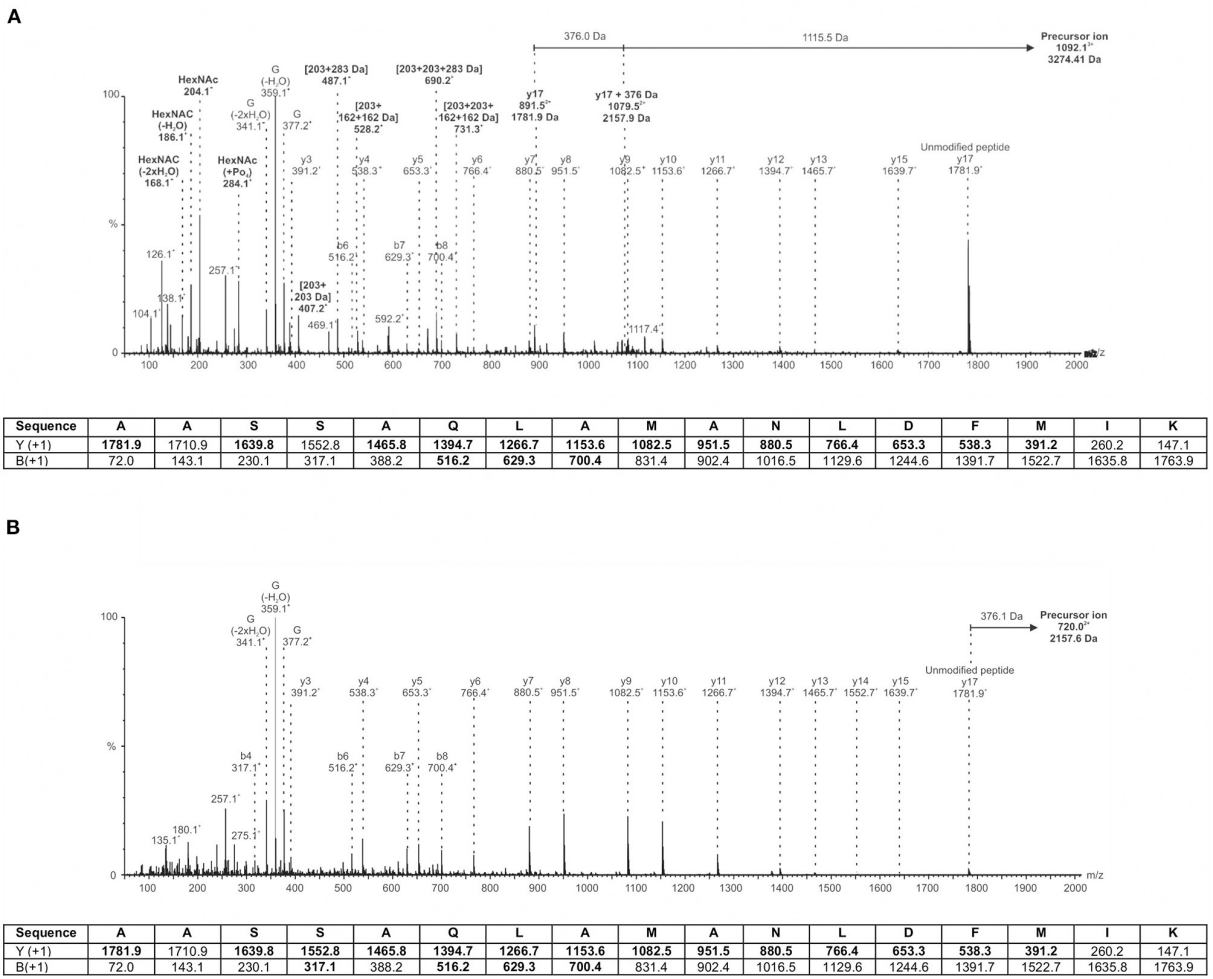
MS/MS spectra of an *A. piscicola* AH-3 polar flagellin glycopeptide. **(A)** Wild-type flagellin is modified by a heterogenous heptasaccharide containing a pseudaminic acid derivative, hexoses, HexNAc with variable phosphorylation and methylation, and an unknown moiety of 102 Da, as observed previously (Wilhelms et al., 2012). Depicted is a representative spectrum of the T18 peptide ^207^AASSAQLAMANLDFMIK^223^ harboring a 1491 Da heptasaccharide glycan that is comprised of a pseudaminic acid derivative of mass 376.14 Da (G) along with two hexoses, two N-acetylhexosamines, a phosphorylated N-actecylhexosamine, and the unknown 102 Da moiety. **(B)** AH-3**Δ**Fgi-1 mutant flagellin is modified by a single monosaccharide only. Depicted is a representative spectrum of the T18 peptide ^207^AASSAQLAMANLDFMIK^223^ harboring the 376 Da pseudaminic acid derivative only. Ions indicating carbohydrate related ions in wild-type **(A)** that are not observed in the glycosyltransferase mutant **(B)** are denoted in bold text. The table below each spectrum indicates the predicted peptide type y and b fragment ions, with those detected indicated in bold.

Our previous work described the normal polar flagellin glycosylation of *A. piscicola* AH3 (Wilhelms et al., 2012). Several sites of modification with a complex and heterogenous heptasaccharide were observed. The glycan chain consisted of a pseudaminic acid derivative (376 Da) linking sugar, followed by two consecutive hexoses (Hex; 162 Da each), three *N*-acetylhexosamines (HexNAc; 203 Da) with and without a variable number of additional phosphate (+80 Da) and methyl (+14 Da) modifications, and finally an unknown carbohydrate moiety of 102 Da. Figure 9A shows a representative spectrum of the wild type T18 peptide ^207^AASSAQLAMANLDFMIK^223^ of FlaB. In this case, the mass difference between the precursor ion (m/z 1092.1^3+^) and the unmodified peptide ion (m/z 1781.9^+^) is 1491.5 Da. This mass difference combined with the observation of several glycan oxonium ions in the lower m/z range of the spectrum (i.e., ions at 204.1^+^, 284.1^+^, 377.2^+^, 407.2^+^, 487.1^+^, 528.2^+^, 690.2^+^, and 731.3^+^ m/z) indicates that the glycan is in fact a heptasaccharide comprised of the pseudaminic acid derivative, along with two Hex, two HexNAc, a phosphorylated HexNAc, and the unknown 102 Da moiety.

In contrast, deletion of the putative glycosylatransferase *fgi-1* results in a single monosaccharide modification of 376 Da, as shown in Figure 9B. The mass difference between the precursor ion (m/z 720.0^3+^) and the same unmodified FlaB peptide ion (m/z 1781.9^+^) is 376.1 Da. There is a corresponding glycan oxonium ion observed at m/z 377.2. However, there are no ions indicating the presence of Hex, HexNAc (with or without variable phosphorylation and methylation), or the 102 Da moiety. The observation of a single pseudaminic acid derivative modification is consistent for all observed FlaA and FlaB glycopeptides in the AH-3ΔFgi-1 mutant (data not shown).

### Complementation and Cross-Complementation of Homologous Fgi-1 of *Aeromonas*

**Figure 10:**
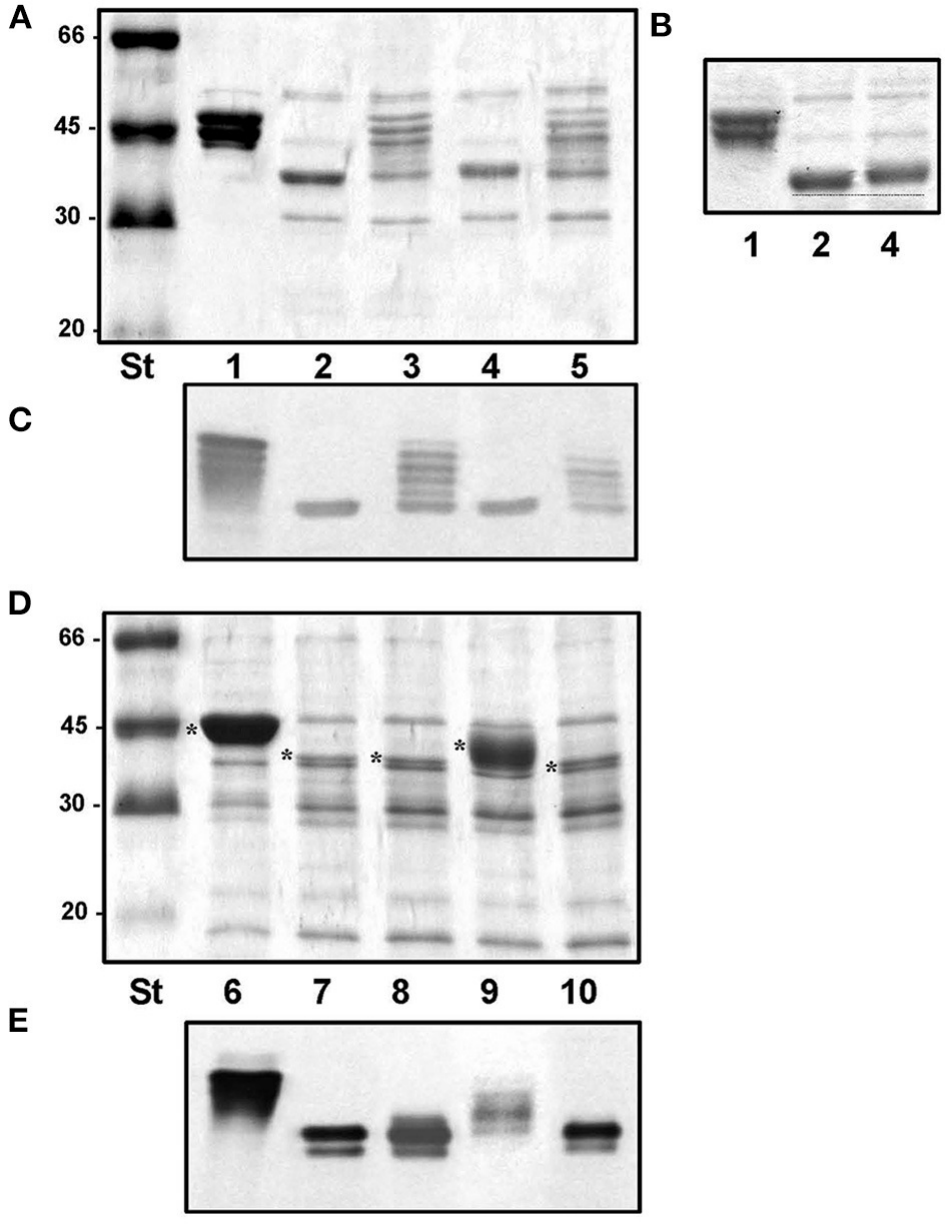
Purified polar flagellum of AH-3 (lane1), AH-3ΔFgi-1 (lane 2), AH-3ΔFgi-1 complemented with the Fgi-1 of *A. piscicola* AH-3 (pBAD33-Fgi-1) (lane 3), with AHA_4171 of *A. hydrophila* (pBAD33-AHA4171) (lane 4) and with M15300 of *A. veronii* (pBAD33-M15300) (lane 5) analyzed using 12% SDS-PAGE **(A)**. Analyzed using 7.5% SDS-PAGE **(B)**. Western blot using *A. piscicola* anti-AH-3 polar flagella antiserum (1:1,000) **(C)**. The dotted line shows the difference in molecular weight between AH-3ΔFgi-1 and AH-3ΔFgi-1 complemented with pBAD33-AHA4171. Size standard (St). Purified polar flagellum from ATCC7966^T^ (lane 6), ATCCΔAHA4171 (lane 7) and ATCCΔAHA4171 complemented with pBAD33-Fgi-1 (lane 8), pBAD33-AHA4171 (lane 9) and pBAD33-M15300 (lane 10) analyzed using 12% SDS-PAGE **(D)**. Western blot using *A. piscicola* anti-AH-3 polar flagella antiserum (1:1,000) **(E)**. Size standard (St). Polar flagellins (*).

To determine whether homologous Fgi-1 are able to complement the AH-3ΔFgi*-*1 and ATCCΔAHA4171 mutants, we cloned the Fgi-1 of *A. piscicola* AH-3 (pBAD33-Fgi-1), the AHA_4171 of *A. hydrophila* ATCC7966^T^ (pBAD33-AHA4171) and the M15300 of *A. veronii* Hm21 (pBAD33-M15300). Fgi-1 of *A. piscicola* AH-3 and M15300 of *A. veronii* Hm21 are included in the Fgi-1 group 1, while AHA_4171 of *A. hydrophila* ATCC7966^T^ is included in the Fgi-1 group 3. Complementation and cross-complementation assays showed that the molecular weight of the polar flagellin of AH-3ΔFgi*-*1 mutant was increased significantly when Fgi-1 of *A. piscicola* AH-3 or M15300 of *A. veronii* Hm21 were introduced, suggesting that the heteroglycan is transferred to polar flagellin (Figures 10A,C). Both complemented strains show similar polar flagellin profiles and have a molecular weight identical to the wild-type AH-3 flagellin. However, cross-complementation of AH-3ΔFgi-1 mutant with AHA_4171 of *A. hydrophila* ATCC7966^T^ only slightly increased the polar flagellin molecular weight (Figures 10A,B), suggesting that this glycosyltransferase was able to link some sugars to the pseudaminic acid derivative on the polar flagellin. When ATCCΔAHA4171 mutant was complemented and cross-complemented, only its own glycosyltransferase was able to increase the polar flagellin molecular weight back to the molecular weight of the wild type, while *trans*-complementation only showed a partial complementation of the flagellin molecular weight (Figures 10D,E).

## Discussion

Glycosylation of secreted proteins is a crucial co- and post-translational modification not only with structural roles but also involved in intrinsic organism intercellular communication, as well as in host-pathogen and symbiont interactions. Glycans and glycoproteins have been widely used as a self/non-self and immune recognition mechanism throughout evolution. Examples include the ABO blood types in humans, vertebrate toll-like receptors (Barton and Medzhitov, 2003), lectins in animals and plants, and the bacteriophage tail spike proteins in viruses (Broeker and Barbirz, 2017). It has been recently reported that glycosylation of extracellular proteins is also common in Gram-negative bacteria, such as *Aeromonadaceae* and *Enterobacteriaceae*, and that the glycans involved in this process have functional roles and immunogenic properties (De Maayer and Cowan, 2016).

Mesophilic *Aeromonas* possess a constitutive and *O-*glycosylated polar flagellum, whose glycan composition ranges from a single pseudaminic acid derivative to a heteropolysaccharide (Canals et al., 2007; Tabei et al., 2009). The extended glycan has been reported to attach to the flagellin monomers via a pseudaminic acid derivative linking sugar and its presence is structurally required for the assembly and function of the polar flagellum filament (Canals et al., 2007). Glycosylation of flagellin has been shown to be essential for flagellar filament assembly in other bacteria as well, with elimination of glycosylation having a significant impact on motility which is a known virulence factor in host-pathogen interactions. For example, in *Clostridium difficile*, disruption of the flagellin glycosyltransferase CD0240 resulted in improperly formed flagellar filaments and a significant reduction in bacterial motility by stab agar assay (Twine et al., 2009). Similar observations have been made for *Campylobacter jejuni* (Goon et al., 2003), *Helicobacter pylori* (Ménard et al., 2014), and *Listeria monocytogenes* (Schirm et al., 2004), when key glycosyltransferase enzymes are genetically disrupted.

In this report, we looked at the genetic basis of *O*-glycosylation of polar flagellin in *Aeromonas*, exploring the different genomic loci that are responsible for the extensive diversity of glycans which modify polar flagellins. In 98 mesophilic *Aeromonas* strains, including *A. piscicola* AH3 and *A. hydrophila* ATCC7966^T^, we identified homologs of known pseudaminic acid biosynthetic genes *pseBCFGIH* (known as *flm* and *neu* in some *Aeromonas*) distributed in two chromosomal locations. It is known that disruption of the *pse* genes completely eliminates lateral and polar flagellin glycosylation in *A. piscicola* AH3, which consequently abolishes flagellar filament formation and bacterial motility (Wilhelms et al., 2012). Here, we have further investigated the genomic fragment of *A. piscicola* AH3 that lies between the *pseBC* and *pseFGIH* genes to better characterize the genetic basis of polar flagellin glycosylation. This region encodes for several glycosyltransferase homologs, and deletion of these genes (*fgi-1* to *fgi-12*) leads to partial disruption of polar flagellin glycosylation in *A. piscicola* AH3, but do not affect lateral flagellin glycosylation. This is indicated by the reduced molecular weight of the polar flagellin proteins and a corresponding reduction of bacterial motility. This suggests that enzymes within this region are involved in the synthesis of carbohydrates or assembly of the glycan beyond the linking sugar. By contrast, *Aeromonas* strains whose polar flagellin glycan has only a single pseudaminic acid derivative to begin with, such as *A. hydrophila* AH-1 and *A. caviae* Sch3N (Tabei et al., 2009; Fulton et al., 2015), do not contain glycosyltransferase homolog genes between the pseudaminic acid biosynthetic genes. Collectively, these data suggest that the Pse sugar is essential for assembly of the flagellar filament and that full glycosylation mediated by glycosyltransferases within *pse* genes are required for full glycosylation and consequently, flagella filament stability and its full motility.

The bioinformatic analysis of 265 mesophilic *Aeromonas* genomes retrieved from Genbank shows that *A. media* WS and *A. veronii* AVNIH1 complete genomes do not possess *pse* homologous genes, which are essential for pseudaminic acid biosynthesis. This suggests that their polar flagella are probably not modified with a pseudaminic acid derivative. Furthermore, in 194 genomes examined, the genes putatively related to polar flagellin glycosylation are clustered in highly polymorphic genomic islands with low average GC content and flanked by pseudaminic acid biosynthetic genes. All of these polar flagella glycosylation islands (FGIs) contain *pseBCFI* genes, with the presence of *pseG* and *pseH* being variable. This variability is produced because the glycosyltransferase domain of PseG and the RimL-like acetyltransferase domain of PseH can be found in the same protein as described in *A. caviae* Sch3N (Tabei et al., 2009). Therefore, strains with only one of these genes may have a chimeric protein with both domains, although a more thorough analysis of all these proteins would be required to confirm this hypothesis. Nevertheless, the presence of *pse* genes and a gene homolog of *maf-1*, which encodes a motility-accessory factor, adjacent to the polar flagella region 2, suggest that most mesophilic *Aeromonas* strains have polar flagellins modified by at least one pseudaminic acid derivative. Maf proteins are considered as candidate transferase enzymes in *Campylobacter jejuni* (Karlyshev et al., 2002) and, in this regard, Maf-1 of *A. caviae* Sch3N, has been shown to be responsible for the transfer of pseudaminic acid to the polar flagellins prior to secretion through the flagellar type III secretion system (Parker et al., 2012).

*Aeromonas* FGIs are highly polymorphic but the genetic organization of their homologous genes allows us to classify them in three groups. Groups I and III are small genomic glycosylation islands almost exclusively containing the *pse* genes and some genes involved in the transference or modification of functional groups, such as deacetylases, reductases, and methyl- and amino- transferases. Group II is a large genomic island containing different glycosyltransferases and proteins involved in fatty acid synthesis, located between the *pse* genes. *A. piscicola* AH3 and *A. hydrophila* ATCC7966^T^ belong to this group.

The majority of aeromonads, when classified using the described FGIs, fall into Groups I and II, with only 16 strains classified in Group III. Strains of *A. hydrophila, A. caviae* and *A. veronii* are broadly distributed across all three groups, although they show a different subgroup distribution. Thus, while FGIs of most *A. hydrophila* strains belong to subgroups IA and IIB, *A. veronii* strains belong to subgroup IIA, and *A. caviae* strains to subgroup IB. FGIs of *A. media* strains belong to group I or III, and *A. jandaei* to group I or II. FGIs of other mesophilic *Aeromonas* species only belong to one FGI group. In particular, FGIs of 12 species only belong to Group I (*A. allosaccharophila* and *A. cavernicola* to IA; *A. aquatic, A. encheleia, A. eucrenophila, A. lusitana, A. molluscorum, A. rivipollensis* and *A. tecta* to IB; *A. bivalvium* and *A. sobria* to IC; and *A. rivuli* to IG), FGIs of five species only belong to Group II (*A. piscicola* and *A. schubertii* to IIA; *A. dhakensis* to IIB; and *A. bestiarum* and *A. enteropelogenes* to IIA and B), and FGIs of two species only belong to Group III (*A. australiensis* to IIIA; and *A. simiae* to IIIB).

As described in *C. jejuni, C. coli* and *P. aeruginosa* (Arora et al., 2001; Szymanski et al., 2003), most *Aeromonas* FGIs are located downstream of their glycosylation target, the polar flagellins. However, while all Group I FGIs are found downstream of the polar flagella region 2, some Group II (IIB) and III (IIIB) FGIs are not closely located to the genes encoding flagellar structural proteins. Group IIIB FGIs were chromosomally located adjacent to homologs of genes thought to be involved in *O*-antigen biosynthesis. This genomic distribution suggests that Group IIB and IIIB FGIs could be involved in the transfer of glycans to different bacterial structures. This has been described in *A. caviae* Sch3N, whose FGI is involved in both polar flagella glycosylation and *O*-antigen biosynthesis, using different and specific transferases (Tabei et al., 2009).

The genomic distribution of *pse* genes in Group I and II FGIs is compared with that observed in Group III. While all FGI groups show the *pseBC* genes at one end, Groups I and II are flanked by *pseI* gene at the other end, and Group II by *pseF*. Interestingly, Group III contains a homolog of *neuC*, which encodes an ortholog of UDP-N-acetylglucosamine 2-epimerase, involved in sialic acid synthesis (Ringenberg et al., 2003). NeuC initiates the biosynthesis of CMP-sialic acid from UDP-N-acetylglucosamine and, given the similarity between the enzymes involved in different nonulosonic acid biosynthetic pathways, strains with FGIs of Group III might contain sialic acid in addition to pseudaminic acid modifying the polar flagellins.

All mesophilic Aeromonas genomes with FGIs show, downstream of the polar flagella region 2, a motility accessory factor gene homologous to *maf-*1 of *A. caviae* Sch3N. The encoded protein is responsible for the transfer of pseudaminic acid to the polar flagellins (Parker et al., 2012, 2014). However, all the strains with Group II FGIs have a second motility accessory factor *(maf-2*) adjacent to the *pseHGI* genes, whose function has not been described. Therefore, a relatively small FGI, containing *pse* homologs and a unique *maf* gene downstream of the polar flagellins (Group I or III), suggest that flagellin proteins of strains are modified with a single nonulosonic acid. An example of this has been reported in *A. hydrophila* AH-1 (Fulton et al., 2015). In contrast, the presence of an FGI with several glycosyltransferases between *pse* genes (Group II) and a second motility accessory factor closely linked to the FGIs, could be related to the biosynthesis of heteropolysaccharidic glycans observed to modify polar flagellins, as described in *A. piscicola* AH-3 (Wilhelms et al., 2012). Furthermore, the presence of gene homologs encoding proteins putatively involved in fatty acid synthesis suggests that polar flagellin glycans may incorporate a liposugar component in strains with FGIs of Group II.

Although *pse* genes, *maf-2* and the genes encoding proteins involved in fatty acid synthesis are highly conserved in strains with Group II FGIs, the genetic variability of the chromosomal fragment between *luxC* and *pseC* is very high. This fragment contains genes encoding different glycosyltransferases, enzymes involved in the transference or modification of functional groups and, in some strains, also sugar biosynthetic genes and transposases. This suggests that the presence of several different glycosyltransferases in this genomic region dictates the number of carbohydrates of the polar flagella glycan. Furthermore, except for *A. caviae* Aer268, all Group II strains show a gene downstream of *luxC* encoding a conserved glycosyltransferase with a GT-2 domain and a GT-A type structural fold that we named Fgi-1 in *A. piscicola* AH-3. In-frame mutants of Fgi-1 and its homologous protein in *A. hydrophila* ATCC7966^T^, AHA_4171, showed a reduction in the amount of polar flagellins assembled in the bacterial surface. Furthermore, the polar flagellins in these mutant strains also had lower molecular weights as compared to their respective wild-type strains, and compromised swimming motility. Furthermore, AH-3ΔFgi*-*1 polar flagellins show the same molecular weight as those of the AH-3ΔFgi mutant. Mass spectrometry analysis of glycopeptides confirms that AH-3ΔFgi-1 polar flagellin is modified by a single O-linked pseudaminic acid derivative linking sugar whereas the wildtype is modified by the heptasaccharide that includes a pseudaminic acid linking sugar. These data suggest that this conserved glycosyltransferase is an important enzyme for the polar flagellin glycosylation in *Aeromonas* strains with type II FGIs, as well as Fgi-1 and homologous proteins found in many Aeromonads could be involved in the transfer of additional sugars to the pseudaminic acid derivative linked to the polar flagellins.

The alignment of Fgi-1 homologous proteins shows a highly conserved N-terminal sequence. This region corresponds to the Rossmann-type nucleotide-binding domain that is terminated by the DxD motif. This motif, as well as the requirement of a divalent cation for activity, is a general feature of the GT-A family of proteins (Breton and Imberty, 1999). Analysis of Fgi-1 of *A. piscicola* AH-3, using the 3DLigandSite server, shows several possible binding sites with Mg and Cu ions in the most significant protein pocket, being Asp94, into the DxD motif, and Tyr215 the interacting residues. The C-terminal region of the protein shows more variability, as described in GT-A glycosyltransferases, and we classified the Fgi-1 homologous proteins in three groups with more than 80% intragroup identity and 55 to 66% intergroup identity. Cross-complementation assays with Fgi-1 homologous proteins, such as M15300 of *A. veronii* Hm21 and AHA_4171 of *A. hydrophila* ATCC7966T, show that only glycosyltransferases belonging to the same group are able to fully restore the AH-3ΔFgi-1 and ATCCΔAHA4171 mutants to their respective wild-type phenotypes. Thus, while Fgi-1 of *A. piscicola* AH-3 and M15300 of *A. veronii* Hm21 are able to fully restore the AH-3 phenotype in the AH-3ΔFgi-1 mutant, AHA_4171 of *A. hydrophila* ATCC7966^T^ only slightly increases the polar flagellin molecular weight of this mutant. In a similar way, only AHA_4171 of *A. hydrophila* ATCC7966^T^ is able to fully restore the ATCC7966^T^ phenotype in the ATCCΔAHA4171 mutant. Collectively, these data could be related to the conserved C-terminal region of Fgi-1 homologous proteins, given that the C-terminal portion of GT-A glycosyltransferases involved in the recognition of the acceptor and, in some proteins, the region corresponding to β6-α4-α5 contains residues that interact with both the donor and the acceptor sugars (Persson et al., 2001). Taken together, these data suggest that Fgi-1 homologous proteins belonging to different groups could transfer the first sugar to the pseudaminic acid derivative which is linked to the amino acid residue, as observed in the AH-3ΔFgi-1 mutant complemented with the AHA_4171 of *A. hydrophila* ATCC7966^T^ and in ATCCΔAHA4171 complemented with the Fgi-1 of *A. piscicola* AH-3. However, the heteropolysaccharidic glycan cannot be built, perhaps because the sugar transferred is not identical to that found in the parental strain or because its linkage with the pseudaminic acid derivative is different and cannot be recognized by other glycosyltransferases involved in the construction of the glycan. Further research is needed to clarify this question.

In the context of host-pathogen interactions, adherence for colonization and infection is an essential step in bacterial pathogenesis. In many motile pathogenic bacteria, flagellin glycosylation can be considered a key virulence factor as it is often required for proper flagella filament assembly and stability, immune evasion, and adhesion to specific host receptors (Twine et al., 2009; Asakura et al., 2010; Nothaft and Szymanski, 2010; Merino and Tomás, 2014). Therefore, the mechanisms of flagellin glycosylation may represent a novel target for antimicrobial development (Fulton et al., 2016) and as such, understanding the genetic factors responsible will continue to be important.

## Data Availability Statement

The original contributions presented in the study are included in the article/Supplementary Material, further inquiries can be directed to the corresponding author/s.

## Author Contributions

SM and JT conceived the project. GF-C and SM performed the genome analyses and conducted the bioinformatics analyses. KF, JS, and ST performed the mass spectrometry analysis. EM-B and SM constructed the defined mutants and plasmid. EM-B and JT performed and analyzed the cross-complementation assays. SM, KF, and ST prepared the manuscript. All authors read and approved the final manuscript. All authors contributed to the article and approved the submitted version.

## Conflict of Interest

The authors declare that the research was conducted in the absence of any commercial or financial relationships that could be construed as a potential conflict of interest.
