# Supplementary material for: Polar Flagella Glycosylation in Aeromonas: Genomic Characterization and Involvement of a Specific Glycosyltransferase (Fgi-1) in Heterogeneous Flagella Glycosylation
Source: Front Microbiol. 2021 Jan 18;11:595697. doi: 10.3389/fmicb.2020.595697 (PMC7874193; doi:10.3389/fmicb.2020.595697)
Supplement: Supplementary file 1 [file Table_1.DOCX]

Supplementary Material

# Supplementary Tables

**Supplementary Table 1.** Classification of mesophilic *Aeromonas* strains with polar flagella glycosylation islands.

| **GROUP I** | | | | |  |
| --- | --- | --- | --- | --- | --- |
| **Subgroup** | **Specie** | **Strain** | | **BioProject** |  |
| IA | *A. allosaccharophila* | BVH88 | | [PRJEB7045](https://www.ncbi.nlm.nih.gov/bioproject/PRJEB7045) |  |
|  |  | CCM4363 | | [PRJNA345312](https://www.ncbi.nlm.nih.gov/bioproject/PRJNA345312) |  |
|  |  | CECT 4199 | | [PRJEB7019](https://www.ncbi.nlm.nih.gov/bioproject/PRJEB7019) |  |
|  |  | TTU2014-159ASC | | [PRJNA296464](https://www.ncbi.nlm.nih.gov/bioproject/PRJNA296464) |  |
|  | *A. cavernicola* | MDC2508 | | [PRJNA417251](https://www.ncbi.nlm.nih.gov/bioproject/PRJNA417251) |  |
|  | *A. hydrophila* | 2JBN101 | | [PRJNA320461](https://www.ncbi.nlm.nih.gov/bioproject/PRJNA320461) |  |
|  |  | 4LNG101 | | [PRJNA343741](https://www.ncbi.nlm.nih.gov/bioproject/PRJNA343741) |  |
|  |  | AH-1 | | [PRJNA323709](https://www.ncbi.nlm.nih.gov/bioproject/PRJNA323709) |  |
|  |  | AH10 | | [PRJNA278509](https://www.ncbi.nlm.nih.gov/bioproject/PRJNA278509) |  |
|  |  | Ah DBHS101 | | [PRJNA379721](https://www.ncbi.nlm.nih.gov/bioproject/PRJNA379721) |  |
|  |  | AL09_71 | | [PRJNA227037](https://www.ncbi.nlm.nih.gov/bioproject/PRJNA227037) |  |
|  |  | AL09-79 | | [PRJNA302467](https://www.ncbi.nlm.nih.gov/bioproject/PRJNA302467) |  |
|  |  | AL10-121 | | [PRJNA302455](https://www.ncbi.nlm.nih.gov/bioproject/PRJNA302455) |  |
|  |  | AL97-91 | | [PRJNA321178](https://www.ncbi.nlm.nih.gov/bioproject/PRJNA321178) |  |
|  |  | Arkansas 2010 | | [PRJNA321184](https://www.ncbi.nlm.nih.gov/bioproject/PRJNA321184) |  |
|  |  | BSK-10 | | [PRJNA381330](https://www.ncbi.nlm.nih.gov/bioproject/PRJNA381330) |  |
|  |  | D4 | | [PRJNA308632](https://www.ncbi.nlm.nih.gov/bioproject/PRJNA308632) |  |
|  |  | FDAARGOS_78 | | [PRJNA231221](https://www.ncbi.nlm.nih.gov/bioproject/PRJNA231221) |  |
|  |  | GYK1 | | [PRJNA323754](https://www.ncbi.nlm.nih.gov/bioproject/PRJNA323754) |  |
|  |  | HZAUAH | | [PRJNA356344](https://www.ncbi.nlm.nih.gov/bioproject/PRJNA356344) |  |
|  |  | J-1 | | [PRJNA227242](https://www.ncbi.nlm.nih.gov/bioproject/PRJNA227242) |  |
|  |  | JBN2301 | | [PRJNA302121](https://www.ncbi.nlm.nih.gov/bioproject/PRJNA302121) |  |
|  |  | ML09_119 | | [PRJNA188141](https://www.ncbi.nlm.nih.gov/bioproject/PRJNA188141) |  |
|  |  | ML09-121 | | [PRJNA302468](https://www.ncbi.nlm.nih.gov/bioproject/PRJNA302468) |  |
|  |  | ML09-122 | | [PRJNA302469](https://www.ncbi.nlm.nih.gov/bioproject/PRJNA302469) |  |
|  |  | MN98-04 | | [PRJNA321182](https://www.ncbi.nlm.nih.gov/bioproject/PRJNA321182) |  |
|  |  | NF1 | | [PRJNA237913](https://www.ncbi.nlm.nih.gov/bioproject/PRJNA237913) |  |
|  |  | NF2 | | [PRJNA237917](https://www.ncbi.nlm.nih.gov/bioproject/PRJNA237917) |  |
|  |  | NJ-35 | | [PRJNA226230](https://www.ncbi.nlm.nih.gov/bioproject/PRJNA226230) |  |
|  |  | pc104A | | [PRJNA227038](https://www.ncbi.nlm.nih.gov/bioproject/PRJNA227038) |  |
|  |  | RB-AH | | [PRJNA253773](https://www.ncbi.nlm.nih.gov/bioproject/PRJNA253773) |  |
|  |  | TN-97-08 | | [PRJNA301651](https://www.ncbi.nlm.nih.gov/bioproject/PRJNA301651) |  |
|  | *Aeromonas sp.* | HMWF015 | | [PRJNA420393](https://www.ncbi.nlm.nih.gov/bioproject/PRJNA420393) |  |
|  |  | HMWF036 | | [PRJNA420393](https://www.ncbi.nlm.nih.gov/bioproject/PRJNA420393) |  |
|  |  | HMWF017 | | [PRJNA420393](https://www.ncbi.nlm.nih.gov/bioproject/PRJNA420393) |  |
|  | *A. veronii* | E2102 | | [PRJNA463043](https://www.ncbi.nlm.nih.gov/bioproject/PRJNA463043) |  |
|  |  | MS-18-37 | | [PRJNA504296](https://www.ncbi.nlm.nih.gov/bioproject/PRJNA504296) |  |
|  |  | Z2-7 | | [PRJNA408193](https://www.ncbi.nlm.nih.gov/bioproject/PRJNA408193) |  |
|  |  | ZWY-AV1 | | [PRJNA416949](https://www.ncbi.nlm.nih.gov/bioproject/PRJNA416949) |  |
| IB | *A. aquatica* | AE235 | | [PRJNA260478](https://www.ncbi.nlm.nih.gov/bioproject/PRJNA260478) |  |
|  | *A. caviae* | 8LM | | [PRJNA277314](https://www.ncbi.nlm.nih.gov/bioproject/PRJNA277314) |  |
|  |  | A23 | | [PRJNA287226](https://www.ncbi.nlm.nih.gov/bioproject/PRJNA287226) |  |
|  |  | BWH65 | | [PRJNA271899](https://www.ncbi.nlm.nih.gov/bioproject/PRJNA271899) |  |
|  |  | CECT838 | | [PRJEB7024](https://www.ncbi.nlm.nih.gov/bioproject/PRJEB7024) |  |
|  |  | CHZ306 | | [PRJNA338160](https://www.ncbi.nlm.nih.gov/bioproject/PRJNA338160) |  |
|  |  | FDAARGOS_75 | | [PRJNA231221](https://www.ncbi.nlm.nih.gov/bioproject/PRJNA231221) |  |
|  |  | HAMBI_1972 | | [PRJNA476209](https://www.ncbi.nlm.nih.gov/bioproject/PRJNA476209) |  |
|  |  | NCTC12244 | | [PRJEB6403](https://www.ncbi.nlm.nih.gov/bioproject/PRJEB6403) |  |
|  |  | R25-6 | | [PRJNA428427](https://www.ncbi.nlm.nih.gov/bioproject/PRJNA428427) |  |
|  |  | YL12 | | [PRJNA242225](https://www.ncbi.nlm.nih.gov/bioproject/PRJNA242225) |  |
|  | *A. encheleia* | CECT4342 | | [PRJEB7027](https://www.ncbi.nlm.nih.gov/bioproject/PRJEB7027) |  |
|  |  | NCTC12917 | | [PRJEB6403](https://www.ncbi.nlm.nih.gov/bioproject/PRJEB6403) |  |
|  | *A. eucrenophila* | CECT4224 | | [PRJEB7029](https://www.ncbi.nlm.nih.gov/bioproject/PRJEB7029) |  |
|  | *A. hydrophila* | 4AK4 | | [PRJNA210524](https://www.ncbi.nlm.nih.gov/bioproject/PRJNA210524) |  |
|  |  | ZYAH72 | | [PRJNA339368](https://www.ncbi.nlm.nih.gov/bioproject/PRJNA339368) |  |
|  | *A. lusitana* | MDC 2473 | | [PRJNA417247](https://www.ncbi.nlm.nih.gov/bioproject/PRJNA417247) |  |
|  | *A. media* | Z1-6 | | [PRJEB27351](https://www.ncbi.nlm.nih.gov/bioproject/PRJEB27351) |  |
|  | *A. molluscorum* | 848 | | [PRJNA183610](https://www.ncbi.nlm.nih.gov/bioproject/PRJNA183610) |  |
|  | *A. rivipollensis* | KN-Mc-11N1 | | [PRJNA438570](https://www.ncbi.nlm.nih.gov/bioproject/PRJNA438570) |  |
|  | *Aeromonas sp.* | 62-46 | | [PRJNA279279](https://www.ncbi.nlm.nih.gov/bioproject/PRJNA279279) |  |
|  |  | ASNIH1 | | [PRJNA430813](https://www.ncbi.nlm.nih.gov/bioproject/PRJNA430813) |  |
|  |  | ASNIH3 | | [PRJNA430813](https://www.ncbi.nlm.nih.gov/bioproject/PRJNA430813) |  |
|  | *A. tecta* | CECT 7082 | | [PRJEB7042](https://www.ncbi.nlm.nih.gov/bioproject/PRJEB7042) |  |
| IC | *A. bivalvium* | CECT7113 | | [PRJEB7023](https://www.ncbi.nlm.nih.gov/bioproject/PRJEB7023) |  |
|  |  | ZJ19-2 | | [PRJNA408193](https://www.ncbi.nlm.nih.gov/bioproject/PRJNA408193) |  |
|  |  | ZJ20-2 | | [PRJNA408193](https://www.ncbi.nlm.nih.gov/bioproject/PRJNA408193) |  |
|  | *A. caviae* | Aer593 | | [PRJNA506284](https://www.ncbi.nlm.nih.gov/bioproject/PRJNA506284) |  |
|  |  | GEO_47_Up_A | | [PRJNA472583](https://www.ncbi.nlm.nih.gov/bioproject/PRJNA472583) |  |
|  |  | GEO_48_Eff_A | | [PRJNA472583](https://www.ncbi.nlm.nih.gov/bioproject/PRJNA472583) |  |
|  | *A. jandaei* | Aer337 | | [PRJNA506284](https://www.ncbi.nlm.nih.gov/bioproject/PRJNA506284) |  |
|  | *A. sobria* | CECT4245 | | [PRJEB7040](https://www.ncbi.nlm.nih.gov/bioproject/PRJEB7040) |  |
|  | *Aeromonas sp.* | ASNIH2 | | [PRJNA430813](https://www.ncbi.nlm.nih.gov/bioproject/PRJNA430813) |  |
|  | *A. veronii* | 5.28.6 | | [PRJNA396650](https://www.ncbi.nlm.nih.gov/bioproject/PRJNA396650) |  |
|  |  | AVNIH2 | | [PRJNA279618](https://www.ncbi.nlm.nih.gov/bioproject/PRJNA279618) |  |
|  |  | ML09-123 | | [PRJNA431414](https://www.ncbi.nlm.nih.gov/bioproject/PRJNA431414) |  |
|  |  | NS | | [PRJNA396650](https://www.ncbi.nlm.nih.gov/bioproject/PRJNA396650) |  |
|  |  | PDB | | [PRJNA396650](https://www.ncbi.nlm.nih.gov/bioproject/PRJNA396650) |  |
|  |  | TH0426 | | [PRJNA293940](https://www.ncbi.nlm.nih.gov/bioproject/PRJNA293940) |  |
|  |  | VCK | | [PRJNA396650](https://www.ncbi.nlm.nih.gov/bioproject/PRJNA396650) |  |
|  | *A. veronii* bv. sobria | LMG13067 | | [PRJEB7051](https://www.ncbi.nlm.nih.gov/bioproject/PRJEB7051) |  |
| ID | *A. jandaei* | Riv2 | | [PRJNA237126](https://www.ncbi.nlm.nih.gov/bioproject/PRJNA237126) |  |
|  | *A. sobria* | 08005 | | [PRJNA344548](https://www.ncbi.nlm.nih.gov/bioproject/PRJNA344548) |  |
|  | *Aeromonas sp.* | SCS5 | | [PRJNA285016](https://www.ncbi.nlm.nih.gov/bioproject/PRJNA285016) |  |
|  | *A. veronii* | FC951 | | [PRJNA428153](https://www.ncbi.nlm.nih.gov/bioproject/PRJNA428153) |  |
| IE | *A. caviae* | Ae398 | | [PRJEA51677](https://www.ncbi.nlm.nih.gov/bioproject/PRJEA51677) |  |
|  |  | CECT4221 | | [PRJEB7046](https://www.ncbi.nlm.nih.gov/bioproject/PRJEB7046) |  |
|  |  | GEO_39_Eff_A | | [PRJNA472583](https://www.ncbi.nlm.nih.gov/bioproject/PRJNA472583) |  |
|  |  | ZJ66-1 | | [PRJNA408193](https://www.ncbi.nlm.nih.gov/bioproject/PRJNA408193) |  |
|  | *A. veronii* | AER39 | | [PRJNA71513](https://www.ncbi.nlm.nih.gov/bioproject/PRJNA71513) |  |
|  |  | CB51 | | [PRJNA319612](https://www.ncbi.nlm.nih.gov/bioproject/PRJNA319612) |  |
| IF | *A. caviae* | L12 | | [PRJNA270276](https://www.ncbi.nlm.nih.gov/bioproject/PRJNA270276) |  |
|  | *A. veronii* bv. sobria | 312M | | [PRJNA498295](https://www.ncbi.nlm.nih.gov/bioproject/PRJNA498295) |  |
| IG | *A. caviae* | GEO_23_Down_B | | [PRJNA472583](https://www.ncbi.nlm.nih.gov/bioproject/PRJNA472583) |  |
|  | *A. media* | ARB13 | | [PRJNA260228](https://www.ncbi.nlm.nih.gov/bioproject/PRJNA260228) |  |
|  |  | ARB20 | | [PRJNA260227](https://www.ncbi.nlm.nih.gov/bioproject/PRJNA260227) |  |
|  | *A. rivuli* | DSM 22539 | | [PRJEB7035](https://www.ncbi.nlm.nih.gov/bioproject/PRJEB7035) |  |
| **GROUP II** | | | | |  |
| **Subgroup** | **Specie** | **Strain** | **BioProject** | |  |
| IIA | 1. *bestiarum* | GA97-22 | [PRJNA431444](https://www.ncbi.nlm.nih.gov/bioproject/PRJNA431444) | |  |
|  | 1. *caviae* | 429865 | [PRJNA292995](https://www.ncbi.nlm.nih.gov/bioproject/PRJNA292995) | |  |
|  |  | Aer268 | [PRJNA506284](https://www.ncbi.nlm.nih.gov/bioproject/PRJNA506284) | |  |
|  |  | FDAARGOS_72 | [PRJNA231221](https://www.ncbi.nlm.nih.gov/bioproject/PRJNA231221) | |  |
|  |  | FDAARGOS_76 | [PRJNA231221](https://www.ncbi.nlm.nih.gov/bioproject/PRJNA231221) | |  |
|  |  | GSH8M-1 | [PRJDB6962](https://www.ncbi.nlm.nih.gov/bioproject/PRJDB6962) | |  |
|  | 1. *enteropelogenes* | 1999lcr | [PRJNA245216](https://www.ncbi.nlm.nih.gov/bioproject/PRJNA245216) | |  |
|  |  | Aer371 | [PRJNA506284](https://www.ncbi.nlm.nih.gov/bioproject/PRJNA506284) | |  |
|  | 1. *piscicola* | AH-3 | [PRJNA323710](https://www.ncbi.nlm.nih.gov/bioproject/PRJNA323710) | |  |
|  |  | LMG24783 | [PRJEB7033](https://www.ncbi.nlm.nih.gov/bioproject/PRJEB7033) | |  |
|  | *A. schubertii* | ATCC 43700 | [PRJNA304368](https://www.ncbi.nlm.nih.gov/bioproject/PRJNA304368) | |  |
|  |  | WL1483 | [PRJNA297116](https://www.ncbi.nlm.nih.gov/bioproject/PRJNA297116) | |  |
|  | *Aeromonas sp.* | ASNIH7 | [PRJNA430813](https://www.ncbi.nlm.nih.gov/bioproject/PRJNA430813) | |  |
|  | *A. veronii* | ARB3 | [PRJNA260226](https://www.ncbi.nlm.nih.gov/bioproject/PRJNA260226) | |  |
|  |  | Hm21 | [PRJNA205862](https://www.ncbi.nlm.nih.gov/bioproject/PRJNA205862) | |  |
|  |  | MS17-88 | [PRJNA492757](https://www.ncbi.nlm.nih.gov/bioproject/PRJNA492757) | |  |
|  |  | pamvotica | [PRJNA356925](https://www.ncbi.nlm.nih.gov/bioproject/PRJNA356925) | |  |
|  |  | TTU2014-108AME | [PRJNA296464](https://www.ncbi.nlm.nih.gov/bioproject/PRJNA296464) | |  |
|  |  | TTU2014-108ASC | [PRJNA296464](https://www.ncbi.nlm.nih.gov/bioproject/PRJNA296464) | |  |
|  |  | TTU2014-115AME | [PRJNA296464](https://www.ncbi.nlm.nih.gov/bioproject/PRJNA296464) | |  |
|  |  | TTU2014-115ASC | [PRJNA296464](https://www.ncbi.nlm.nih.gov/bioproject/PRJNA296464) | |  |
|  |  | TTU2014-125ASC | [PRJNA296464](https://www.ncbi.nlm.nih.gov/bioproject/PRJNA296464) | |  |
|  |  | TTU2014-130AME | [PRJNA296464](https://www.ncbi.nlm.nih.gov/bioproject/PRJNA296464) | |  |
|  |  | TTU2014-130ASC | [PRJNA296464](https://www.ncbi.nlm.nih.gov/bioproject/PRJNA296464) | |  |
|  |  | TTU2014-134ASC | [PRJNA296464](https://www.ncbi.nlm.nih.gov/bioproject/PRJNA296464) | |  |
|  |  | TTU2014-140ASC | [PRJNA296464](https://www.ncbi.nlm.nih.gov/bioproject/PRJNA296464) | |  |
|  |  | TTU2014-141AME | [PRJNA296464](https://www.ncbi.nlm.nih.gov/bioproject/PRJNA296464) | |  |
|  |  | TTU2014-141ASC | [PRJNA296464](https://www.ncbi.nlm.nih.gov/bioproject/PRJNA296464) | |  |
|  |  | TTU2014-142ASC | [PRJNA296464](https://www.ncbi.nlm.nih.gov/bioproject/PRJNA296464) | |  |
|  |  | TTU2014-143ASC | [PRJNA296464](https://www.ncbi.nlm.nih.gov/bioproject/PRJNA296464) | |  |
|  |  | XH.VA.1 | [PRJNA447880](https://www.ncbi.nlm.nih.gov/bioproject/PRJNA447880) | |  |
|  |  | XH.VA.2 | [PRJNA482711](https://www.ncbi.nlm.nih.gov/bioproject/PRJNA482711) | |  |
| IIB | 1. *bestiarum* | CECT4227 | [PRJEB7022](https://www.ncbi.nlm.nih.gov/bioproject/PRJEB7022) | |  |
|  | 1. *caviae* | R25-2 | [PRJNA428427](https://www.ncbi.nlm.nih.gov/bioproject/PRJNA428427) | |  |
|  |  | T25-39 | [PRJNA428427](https://www.ncbi.nlm.nih.gov/bioproject/PRJNA428427) | |  |
|  | 1. *dhakensis* | 17FW001 | [PRJNA484846](https://www.ncbi.nlm.nih.gov/bioproject/PRJNA484846) | |  |
|  |  | 173 | [PRJNA183198](https://www.ncbi.nlm.nih.gov/bioproject/PRJNA183198) | |  |
|  |  | 277 | [PRJNA183202](https://www.ncbi.nlm.nih.gov/bioproject/PRJNA183202) | |  |
|  |  | AAK1 | [PRJDB70](https://www.ncbi.nlm.nih.gov/bioproject/PRJDB70) | |  |
|  |  | AE-13 | [PRJNA504324](https://www.ncbi.nlm.nih.gov/bioproject/PRJNA504324) | |  |
|  |  | Aer283 | [PRJNA506284](https://www.ncbi.nlm.nih.gov/bioproject/PRJNA506284) | |  |
|  |  | CAIM1873 | [PRJNA422283](https://www.ncbi.nlm.nih.gov/bioproject/PRJNA422283) | |  |
|  |  | CECT7289 | [PRJEB7020](https://www.ncbi.nlm.nih.gov/bioproject/PRJEB7020) | |  |
|  |  | CIP 107500 | [PRJEB7048](https://www.ncbi.nlm.nih.gov/bioproject/PRJEB7048) | |  |
|  |  | Cr1 | [PRJNA281575](https://www.ncbi.nlm.nih.gov/bioproject/PRJNA281575) | |  |
|  |  | Cr2 | [PRJNA281576](https://www.ncbi.nlm.nih.gov/bioproject/PRJNA281576) | |  |
|  |  | F2S2-1 | [PRJNA312130](https://www.ncbi.nlm.nih.gov/bioproject/PRJNA312130) | |  |
|  |  | KN-Mc-6U21 | [PRJNA400818](https://www.ncbi.nlm.nih.gov/bioproject/PRJNA400818) | |  |
|  |  | KOR1 | [PRJNA294992](https://www.ncbi.nlm.nih.gov/bioproject/PRJNA294992) | |  |
|  |  | SSU | [PRJNA71509](https://www.ncbi.nlm.nih.gov/bioproject/PRJNA71509) | |  |
|  | 1. *enteropelogenes* | LK14 | [PRJNA277406](https://www.ncbi.nlm.nih.gov/bioproject/PRJNA277406) | |  |
|  | - *A. hydrophila* | 14 | [PRJNA183195](https://www.ncbi.nlm.nih.gov/bioproject/PRJNA183195) | |  |
|  |  | 116 | [PRJNA183196](https://www.ncbi.nlm.nih.gov/bioproject/PRJNA183196) | |  |
|  |  | 187 | [PRJNA183199](https://www.ncbi.nlm.nih.gov/bioproject/PRJNA183199) | |  |
|  |  | 226 | [PRJNA183200](https://www.ncbi.nlm.nih.gov/bioproject/PRJNA183200) | |  |
|  |  | 259 | [PRJNA183201](https://www.ncbi.nlm.nih.gov/bioproject/PRJNA183201) | |  |
|  |  | 48_AHYD | [PRJNA267549](https://www.ncbi.nlm.nih.gov/bioproject/PRJNA267549) | |  |
|  |  | 50_AHYD | [PRJNA267549](https://www.ncbi.nlm.nih.gov/bioproject/PRJNA267549) | |  |
|  |  | 52_AHYD | [PRJNA267549](https://www.ncbi.nlm.nih.gov/bioproject/PRJNA267549) | |  |
|  |  | 53_AHYD | [PRJNA267549](https://www.ncbi.nlm.nih.gov/bioproject/PRJNA267549) | |  |
|  |  | 56_AHYD | [PRJNA267549](https://www.ncbi.nlm.nih.gov/bioproject/PRJNA267549) | |  |
|  |  | AD9 | [PRJNA236257](https://www.ncbi.nlm.nih.gov/bioproject/PRJNA236257) | |  |
|  |  | Ae34 | [PRJDB1739](https://www.ncbi.nlm.nih.gov/bioproject/PRJDB1739) | |  |
|  |  | AHNIH1 | [PRJNA273636](https://www.ncbi.nlm.nih.gov/bioproject/PRJNA273636) | |  |
|  |  | AHNIH2 | [PRJNA430813](https://www.ncbi.nlm.nih.gov/bioproject/PRJNA430813) | |  |
|  |  | AL06-06 | [PRJNA270887](https://www.ncbi.nlm.nih.gov/bioproject/PRJNA270887) | |  |
|  |  | ATCC7966 | [PRJNA16697](https://www.ncbi.nlm.nih.gov/bioproject/PRJNA16697) | |  |
|  |  | L14f | [PRJNA270277](https://www.ncbi.nlm.nih.gov/bioproject/PRJNA270277) | |  |
|  |  | KN-Mc-1R2 | [PRJNA438415](https://www.ncbi.nlm.nih.gov/bioproject/PRJNA438415) | |  |
|  |  | M013 | [PRJNA264123](https://www.ncbi.nlm.nih.gov/bioproject/PRJNA264123) | |  |
|  |  | M023 | [PRJNA265923](https://www.ncbi.nlm.nih.gov/bioproject/PRJNA265923) | |  |
|  |  | M052 | [PRJNA326907](https://www.ncbi.nlm.nih.gov/bioproject/PRJNA326907) | |  |
|  |  | M053 | [PRJNA326908](https://www.ncbi.nlm.nih.gov/bioproject/PRJNA326908) | |  |
|  |  | M062 | [PRJNA265924](https://www.ncbi.nlm.nih.gov/bioproject/PRJNA265924) | |  |
|  |  | NCT8049 | [PRJEB6403](https://www.ncbi.nlm.nih.gov/bioproject/PRJEB6403) | |  |
|  |  | RU34A | [PRJEB18852](https://www.ncbi.nlm.nih.gov/bioproject/PRJEB18852) | |  |
|  |  | SNUFPC-A8 | [PRJNA175471](https://www.ncbi.nlm.nih.gov/bioproject/PRJNA175471) | |  |
|  |  | TPS-30 | [PRJNA381370](https://www.ncbi.nlm.nih.gov/bioproject/PRJNA381370) | |  |
|  |  | WCX23 | [PRJNA530076](https://www.ncbi.nlm.nih.gov/bioproject/PRJNA530076) | |  |
|  |  | YL17 | [PRJNA234473](https://www.ncbi.nlm.nih.gov/bioproject/PRJNA234473) | |  |
|  |  | ZYAH75 | [PRJNA339336](https://www.ncbi.nlm.nih.gov/bioproject/PRJNA339336) | |  |
|  | - *A. hydrophila subsp. ranae* | CIP107985 | [PRJEB7049](https://www.ncbi.nlm.nih.gov/bioproject/PRJEB7049) | |  |
|  | 1. *jandaei* | IMETJ | [PRJNA432735](https://www.ncbi.nlm.nih.gov/bioproject/PRJNA432735) | |  |
|  |  | L14h | [PRJNA270278](https://www.ncbi.nlm.nih.gov/bioproject/PRJNA270278) | |  |
|  | *Aeromonas sp.* | ASNIH4 | [PRJNA430813](https://www.ncbi.nlm.nih.gov/bioproject/PRJNA430813) | |  |
|  |  | CA23 | [PRJNA400916](https://www.ncbi.nlm.nih.gov/bioproject/PRJNA400916) | |  |
|  |  | RU34C | [PRJEB18864](https://www.ncbi.nlm.nih.gov/bioproject/PRJEB18864) | |  |
|  |  | RU39B | [PRJEB18855](https://www.ncbi.nlm.nih.gov/bioproject/PRJEB18855) | |  |
|  |  | YN13HZO-058 | [PRJNA354843](https://www.ncbi.nlm.nih.gov/bioproject/PRJNA354843) | |  |
|  | *A. veronii* | 17ISAe | [PRJNA438884](https://www.ncbi.nlm.nih.gov/bioproject/PRJNA438884) | |  |
|  |  | A29 | [PRJNA329585](https://www.ncbi.nlm.nih.gov/bioproject/PRJNA329585) | |  |
|  |  | AMC34 | [PRJNA71515](https://www.ncbi.nlm.nih.gov/bioproject/PRJNA71515) | |  |
|  |  | CIP 107763 | [PRJEB7047](https://www.ncbi.nlm.nih.gov/bioproject/PRJEB7047) | |  |
|  |  | CQ-AV1 | [PRJNA511801](https://www.ncbi.nlm.nih.gov/bioproject/PRJNA511801) | |  |
|  |  | RU31B | [PRJEB18858](https://www.ncbi.nlm.nih.gov/bioproject/PRJEB18858) | |  |
|  |  | X11 | [PRJNA419084](https://www.ncbi.nlm.nih.gov/bioproject/PRJNA419084) | |  |
|  |  | X12 | [PRJNA419086](https://www.ncbi.nlm.nih.gov/bioproject/PRJNA419086) | |  |
|  |  | ZJ12-3 | [PRJNA408193](https://www.ncbi.nlm.nih.gov/bioproject/PRJNA408193) | |  |
| **GROUP III** | | | | |  |
| **Subgroup** | **Specie** | **Strain** | **BioProject** | |  |
| IIIA | *A. australiensis* | CECT8023 | [PRJEB7021](https://www.ncbi.nlm.nih.gov/bioproject/PRJEB7021) | |  |
|  | 1. *caviae* | CH129 | [PRJNA339286](https://www.ncbi.nlm.nih.gov/bioproject/PRJNA339286) | |  |
|  | *Aeromonas sp.* | ASNIH5 | [PRJNA430813](https://www.ncbi.nlm.nih.gov/bioproject/PRJNA430813) | |  |
|  | *A. veronii* | CCM7244 | [PRJNA345309](https://www.ncbi.nlm.nih.gov/bioproject/PRJNA345309) | |  |
|  | 1. *caviae* | VBF856 | [PRJNA320016](https://www.ncbi.nlm.nih.gov/bioproject/PRJNA320016) | |  |
| IIIB | 1. *simiae* | CIP107798 | [PRJEB7039](https://www.ncbi.nlm.nih.gov/bioproject/PRJEB7039) | |  |
|  | *A. veronii* | 126-14 | [PRJNA431081](https://www.ncbi.nlm.nih.gov/bioproject/PRJNA431081) | |  |
|  |  | AER397 | [PRJNA71517](https://www.ncbi.nlm.nih.gov/bioproject/PRJNA71517) | |  |
|  |  | AMC35 | [PRJNA71519](https://www.ncbi.nlm.nih.gov/bioproject/PRJNA71519) | |  |
|  |  | B565 | [PRJNA63671](https://www.ncbi.nlm.nih.gov/bioproject/PRJNA63671) | |  |
|  |  | CCM4359 | [PRJNA345311](https://www.ncbi.nlm.nih.gov/bioproject/PRJNA345311) | |  |
|  |  | CECT4257 | [PRJEB7044](https://www.ncbi.nlm.nih.gov/bioproject/PRJEB7044) | |  |
| IIIC | *A. hydrophila* | MX16A | [PRJNA353572](https://www.ncbi.nlm.nih.gov/bioproject/PRJNA353572) | |  |
|  |  | WCHAH045096 | [PRJNA41533](https://www.ncbi.nlm.nih.gov/bioproject/PRJNA415336) | |  |
|  | 1. *media* | S2_003_000_R3_19 | [PRJNA376580](https://www.ncbi.nlm.nih.gov/bioproject/PRJNA376580) | |  |
|  | *Aeromonas sp.* | CU5 | [PRJNA400916](https://www.ncbi.nlm.nih.gov/bioproject/PRJNA400916) | |  |
