# Supplementary material for: Polar Flagella Glycosylation in Aeromonas: Genomic Characterization and Involvement of a Specific Glycosyltransferase (Fgi-1) in Heterogeneous Flagella Glycosylation
Source: Front Microbiol. 2021 Jan 18;11:595697. doi: 10.3389/fmicb.2020.595697 (PMC7874193; doi:10.3389/fmicb.2020.595697)

Supplementary Material

1. Supplementary Figures

Supplementary Figure 1. Alignment performed by ClustalW of the Fgi-1 amino acid sequences of mesophilic *Aeromonas* strains with polar flagella glycosylation islands group II. Fgi-1 groups are separated by brackets and sequences labelled in grey were used in the complementation assays. Black triangles show the Asp94 and Tyr215 interacting residues at the protein pocket.

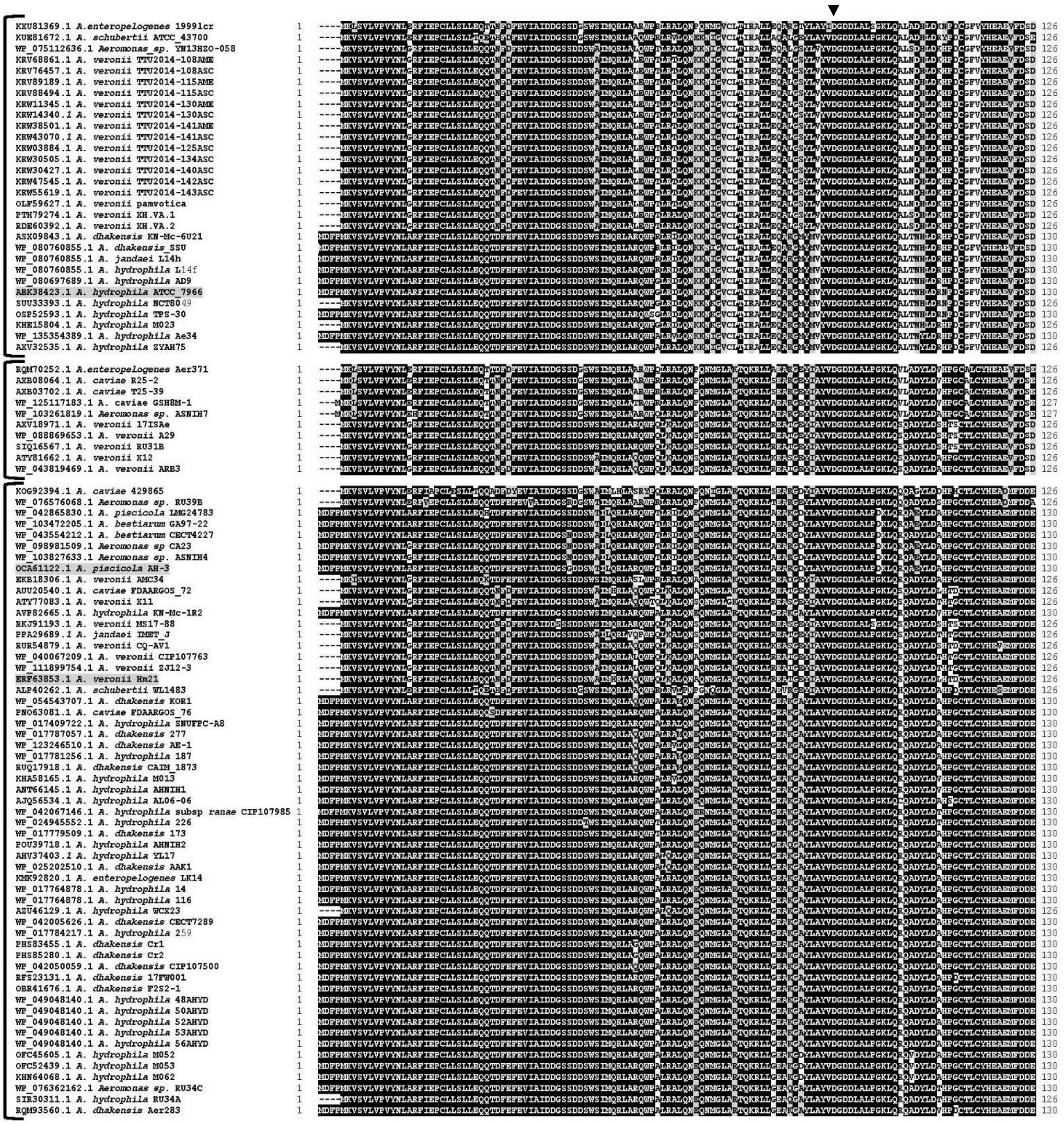

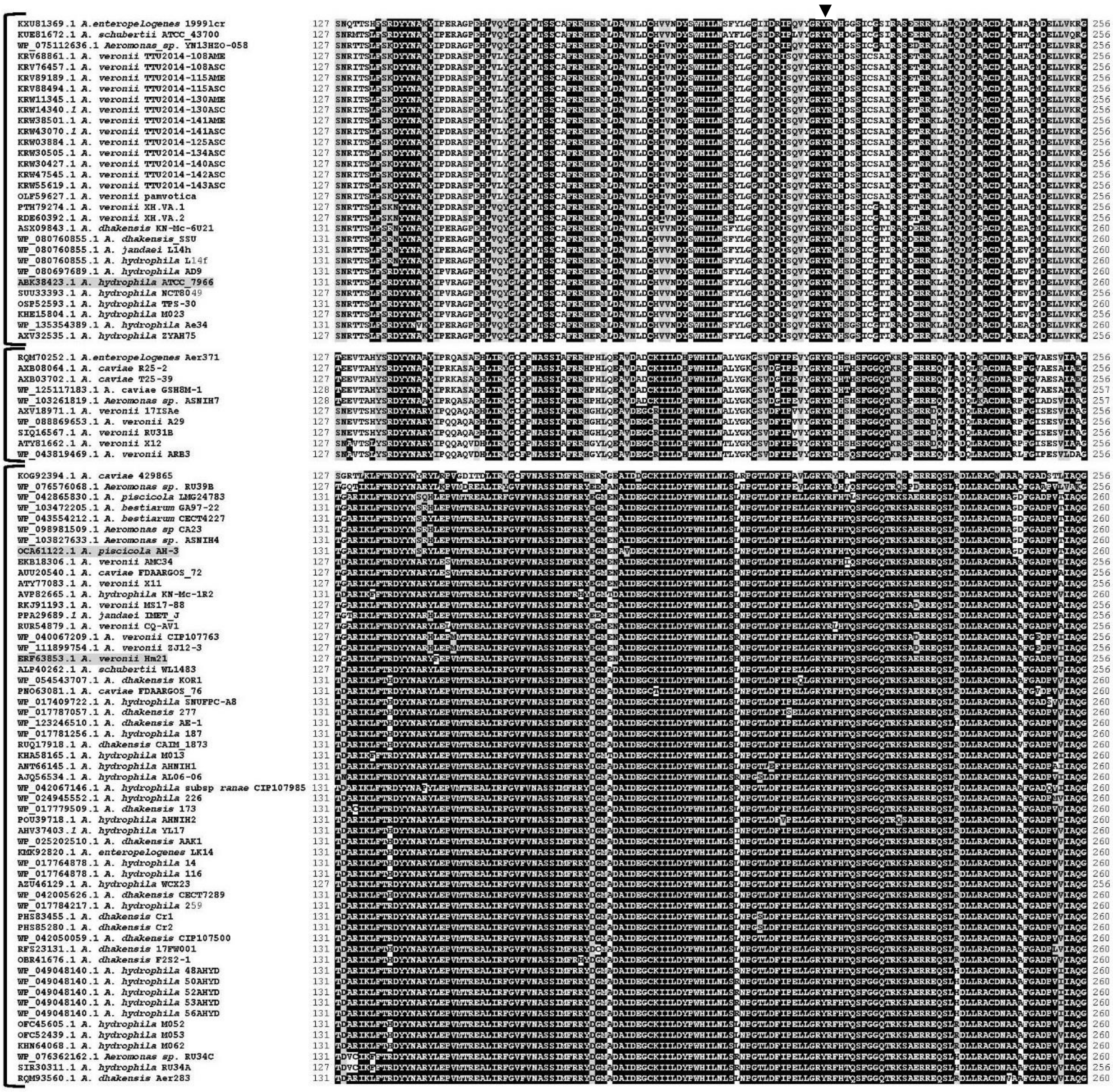

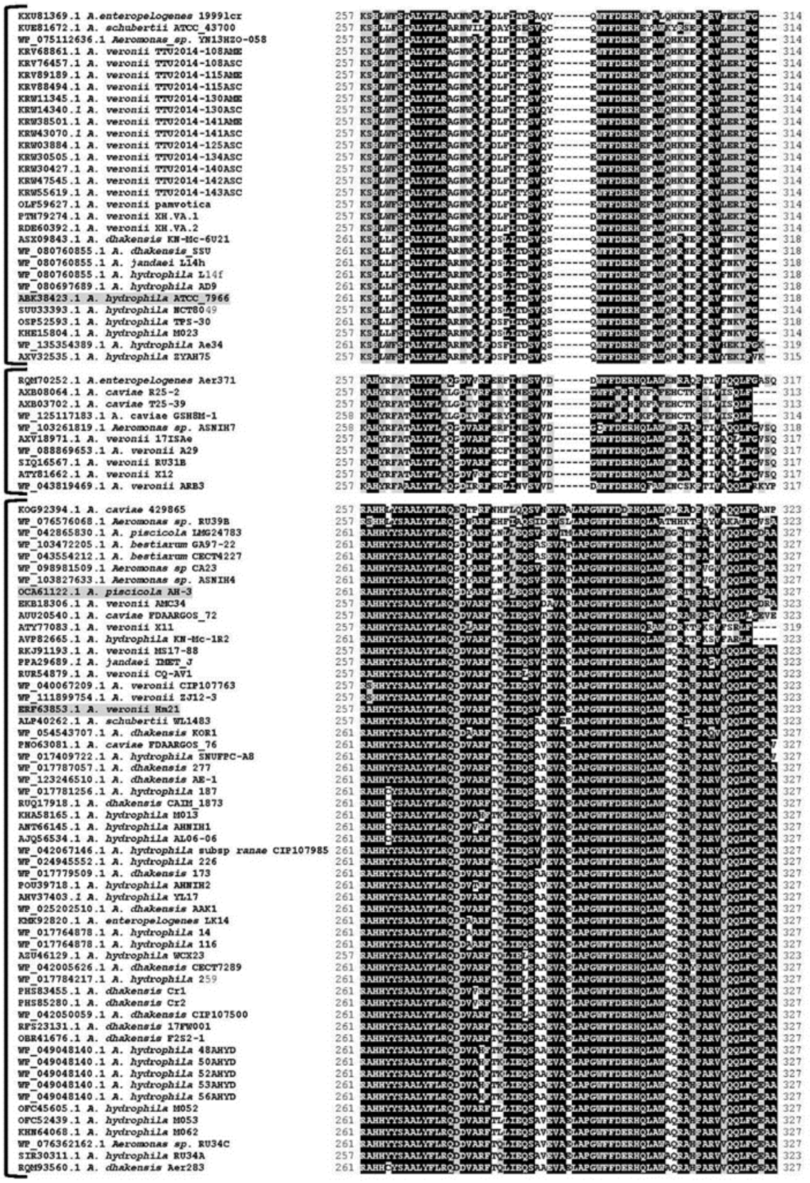

Supplement: Supplementary file 2 [file Image_1.PDF]
